# The Epidermal Microbiome Within an Aggregation of Leopard Sharks (*Triakis semifasciata*) Has Taxonomic Flexibility with Gene Functional Stability Across Three Time-points

**DOI:** 10.1007/s00248-022-01969-y

**Published:** 2022-02-07

**Authors:** Michael P. Doane, Colton J. Johnson, Shaili Johri, Emma N. Kerr, Megan M. Morris, Ric Desantiago, Abigail C. Turnlund, Asha Goodman, Maria Mora, Laís Farias Oliveira Lima, Andrew P. Nosal, Elizabeth A. Dinsdale

**Affiliations:** 1 College of Science and Engineering, Flinders University, Bedford Park, South Australia Australia; 2 Department of Biology, San Diego State University, San Diego, CA USA; 3 Hopkins Marine Station, Stanford University, Pacific Grove, CA USA; 4 Laurence Livermore National Labs, Livermore, CA USA; 5 Australian Centre for Ecogenomics, University of Queensland, St Lucia, QLD Australia; 6 Department of Environmental and Ocean Sciences, University of San Diego, San Diego, CA USA; 7 Scripps Institution of Oceanography, University of California – San Diego, CA La Jolla, USA

**Keywords:** Microbiome, Leopard shark, *Triakis semifasciata*, Shark skin, Next-generation sequencing

## Abstract

The epidermis of Chondrichthyan fishes consists of dermal denticles with production of minimal but protein-rich mucus that collectively, influence the attachment and biofilm development of microbes, facilitating a unique epidermal microbiome. Here, we use metagenomics to provide the taxonomic and functional characterization of the epidermal microbiome of the *Triakis semifasciata* (leopard shark) at three time-points collected across 4 years to identify links between microbial groups and host metabolism. Our aims include (1) describing the variation of microbiome taxa over time and identifying recurrent microbiome members (present across all time-points); (2) investigating the relationship between the recurrent and flexible taxa (those which are not found consistently across time-points); (3) describing the functional compositions of the microbiome which may suggest links with the host metabolism; and (4) identifying whether metabolic processes are shared across microbial genera or are unique to specific taxa. Microbial members of the microbiome showed high similarity between all individuals (Bray–Curtis similarity index = 82.7, where 0 = no overlap, 100 = total overlap) with the relative abundance of those members varying across sampling time-points, suggesting flexibility of taxa in the microbiome. One hundred and eighty-eight genera were identified as recurrent, including *Pseudomonas*, *Erythrobacter*, *Alcanivorax*, *Marinobacter*, and *Sphingopxis* being consistently abundant across time-points, while *Limnobacter* and *Xyella* exhibited switching patterns with high relative abundance in 2013, *Sphingobium* and *Sphingomona* in 2015, and *Altermonas*, *Leeuwenhoekiella*, *Gramella*, and *Maribacter* in 2017. Of the 188 genera identified as recurrent, the top 19 relatively abundant genera formed three recurrent groups. The microbiome also displayed high functional similarity between individuals (Bray–Curtis similarity index = 97.6) with gene function composition remaining consistent across all time-points. These results show that while the presence of microbial genera exhibits consistency across time-points, their abundances do fluctuate. Microbial functions however remain stable across time-points; thus, we suggest the leopard shark microbiomes exhibit functional redundancy. We show coexistence of microbes hosted in elasmobranch microbiomes that encode genes involved in utilizing nitrogen, but not fixing nitrogen, degrading urea, and resistant to heavy metal.

**Supplementary Information:**

The online version contains supplementary material available at 10.1007/s00248-022-01969-y.

## Introduction

The epidermis is the largest organ in the body, and serves as the first line of defense against environmental influences [70]. Along with providing broad protections, the skin of the organism interacts with surrounding microbes and a specific microbiome occurs on different areas of the body and varies between species [1, 28, 32, 92, 98]. In the marine environment, the skin of elasmobranchs and teleost fishes have microbiomes that are distinctive to the surrounding water [26, 58, 81, 89] and a comparison across seven elasmobranchs, including thresher sharks (*Alopias vulpinus*), whale sharks (*Rhincodon typus*), leopard sharks (*Triakis semifasciata*), blacktip reef sharks (*Carcharhinus melanopterus*), nurse shark (*Ginglymostoma cirratum*), lemon shark (*Negaprion brevirostris*), round rays (*Urobatis helleri*), and southern stingrays (*Hypanus americanus*), confirmed that the elasmobranch epidermal microbiomes are species-specific [9, 26, 28, 82]. However, the selection and maintenance of the microbiome on sharks remains an outstanding question, and may be the result of several processes, including, but not limited to, (1) biophysical properties of the epidermis, (2) epidermal secretions of the shark, and (3) interspecific interactions between the microbes.

The biophysical properties of the chondrichthyan fish epidermis are uniquely covered with dermal denticles that improve hydro-dynamism [53]. Dermal denticles have ridges and troughs arranged in overlapping patterns that alter the hydrodynamic properties of water close to the epidermis [90, 99, 103] and this biophysical property potentially affects microbial growth. In synthetic models mimicking shark skin, two bacteria, *Escherichia coli* and *Staphylococcus aureus*, showed differential growth patterns compared to a smooth surface. *E. coli* had high attachment rates to the structured surface, whereas *S. aureus* was not able to attach [13], therefore, suggesting that only specific types of microbes are able to attach to the shark skin. Further, modelling of microbial community dynamics showed that high surface structural complexity (like the skin of sharks) can reduce competition and increase coexistence between microbes [61]. Therefore, the high micro-structured nature of the shark skin surface may produce similar dynamics in the microbiome. The epidermis of Chondrichthyan fish is characterized by a lower level of secretory cells compared with Actinopterygian fishes [70] and the product is a thin heterogeneous outer mucous layer (5–8 μm thickness; pH between 6 and 7). The mucus contains high levels of proteins, with only a few proteins being identified [95] and has moderate to high disulfide concentrations that are suspected to provide mechanical stabilization of the mucus coating [71]. In addition to the physical structure of the dermal denticles, the epidermis is covered in protrusions that may stabilize the thin mucus layer [70] and microvilli-like apical protrusions, which may act as chemoreceptors [80], contributing to the highly structured nature of the skin surface.

The metabolism of elasmobranchs relies on ketone bodies, fatty acids, carbohydrates, and amino acids for energy production [88]. Amino acids are ketogenic precursors, oxidative fuel, and nitrogen suppliers [88]. Nitrogen metabolism supports the production of high amount of urea and trimethylamine N-oxide (TMAO) for osmoregulation [6, 88]. Exocytotic activity is observable where the contents of the mucous vesicles below the apical membrane are transported onto the epidermal surface or into the thin mucus layer [70], suggesting potential processes for the products of host metabolism to be available to the microbiome. Sharks bioaccumulate toxins and heavy metals from lower trophic levels [33, 67], absorb trace metals into their skin at a faster rate than teleost fishes [42–46], and have been shown to exhibit maternal off-loading to their offspring in mucus surrounding the developing embryo (Lyons and Lowe [63]). Therefore, metabolically derived compounds and harmful contaminants are being leached from elasmobranchs into their mucus and may affect the function of the skin microbes. For instance, the microbiome signature from thresher sharks (*Alopias vulinpus*) suggests potential heavy metal concentration at the skin interface signified by high abundance of heavy metal resistance genes [26].

Interactions between the microbes on the surface and those from the surrounding water are also expected to affect the characteristics of the microbiome. The low levels of mucus secreted by the sharks and minimal microbial biofilm development that occurred on the synthetic models of the shark skin, compared with the pronounced biofilm development on the smooth surface [13], suggest that microbial growth on the epidermis is suppressed, and the turnover of microbial communities may be high. Conversely, stability of a microbiome is important for the host’s health and functioning, and rapid or unexpected variations in microbiome taxonomy can lead to a state of dysbiosis, resulting in a decline in organismal health [20, 31, 58]. Therefore, an understanding of how the microbiome varies through time is important, but there are limited investigations of microbiome stability over time in the marine environment. A few examples include the epidermal microbiome of humpback whales (*Megaptera novaeangliae*) and Atlantic cod (*Gadus morhua*) which varied seasonally [8, 101], and similar seasonal changes occurred in the microbiome of the stony coral, *Montastrea faveolata* [51]. While the respiratory microbiome of bottlenose dolphin species (*Tursiops truncatus* and *Tursiops aduncus*) maintained taxonomic stability over 2 months, individual dolphins had significantly different microbiome taxonomic structures [56]. In an analysis over 8 months, captive dolphins had an individual signature of their lung microbiome and shared between 17 and 41% of the microbiome with other individuals [96]. In contrast, 77.6% of the microbes in the epidermal microbiome of thresher sharks (*A. vulpinus*) were shared across individual sharks [26], however, this study was only conducted at one time-point. Therefore, whether the epidermal microbiomes of marine vertebrate hosts are temporally stable or flexible remains an outstanding question in science.

Here we analyze both the taxonomic make-up and functional capabilities of the leopard shark (*Triakis semifasciata*) epidermal microbiome over three time-points measured across 4 years using the following objectives: (1) to describe the variation of microbiome taxa over time and identify those members which are recurrent (present on all individuals across all time-points); (2) to investigate the relationship between the recurrent and flexible taxa (those which are not found consistently across time-points); (3) to describe the functional compositions of the microbiome which may suggest links with the host metabolism; and (4) to identify whether the metabolic processes are shared across microbial genera or are unique to specific taxa.

## Materials and Methods

### Epidermal Microbiome Sampling

Leopard sharks (*Triakis Semifasciata*) form consistent aggregations in late summer, early fall at near-shore locations [76, 77] along the coast of California making this species a good candidate to investigate temporal microbiome dynamics of a wild animal. Sharks in this study were sampled from La Jolla Cove, CA, USA (32° 51′ 12″ N, 117° 15′ 48″ W). Sampled individuals were all females, reflecting previously described sex-specific aggregation behavior exhibited by *T. semifasciata* along the southern California coast [76, 77]. It is suspected that females aggregate in this location because they are pregnant [77], however, pregnancy status cannot be detected visually and requires the use of species-specific hormone analysis and ultra-sound equipment [21], which were beyond the scope of this study. All individuals were > 0.9 m, which is the estimated reproductive age for *T. semifasciata* [19]. We targeted at least three sharks per year and captured seven sharks in 2013, three in 2015, and eight in 2017; thus, the study was conducted at three time-points collected over a 4-year period. While *T. semifasciata* form aggregations, it is not always the same individual sharks that return each year [76]. In addition, *T. semifasciata* are small, and their fins are not strong enough to support a satellite tracker that could be used to pinpoint the position of an individual for recapture. Even if tagging was possible, seeing a tagged shark from the surface and recatching the tagged individual is unlikely. Therefore, recapture of the same individual shark across multiple years was not feasible and we opted to measure the microbiome across different sharks captured across the 4 years. The sharks were caught with a handline on baited barbless circle hooks and brought onboard a small skiff for processing using a scoop net; all sharks were released after processing. Microbiome samples from the sharks were collected using a microbial collection tool, which flushes the epidermis with ~ 250 ml of sterile seawater and dislodges the microbes. The microbial slurry is collected into the back end of the microbial tool, as we have conducted previously [26–28, 47, 55]. Each epidermal microbiome sample was taken from the dorso-lateral region in line with the dorsal fin and above the lateral line. The collected microbiome samples were filtered through a 0.22-µm Sterivex filter (Millipore, Inc.) and stored in a − 20 °C freezer until processing.

### DNA Extraction and Sequencing

Microbiome DNA was extracted using a cell lysis and spin-column filtration method by Macherey Nagel Tissue Kit. The purified DNA was prepared for random shot-gun sequencing using the standard protocol from Swift 2S Plus Kit (Swift Biosciences). The DNA library was sequenced using a Mi-Seq Illumina sequencer with Mi-Seq v3 Reagent Kit. Samples were bar-coded and the *T. semifasciata* microbiomes were mixed in with a range of microbiome samples (e.g., kelp, fish, and seagrass microbiome samples) and run on several sequencing runs by the undergraduates in San Diego State University ecological metagenomics class [30].

### Metagenome Annotation

The sequenced metagenomes were processed through PRINSEQ ±  ± software for quality control, removing sequences less than 70 bp, those that had a single N (ambiguous base call), or a quality score below 25. MID tags were also removed with PRINSEQ ++ [11, 86]. The resulting two fasta files were paired using FLASH software [64]. After pairing, metagenomes were uploaded into MG-RAST Version 4.0.3, which provided taxonomic and functional gene annotations of the metagenomes using standard cut-offs [5, 69]. The sequences with the highest bit score were reported as we have done previously [39, 73, 75, 97]. The taxonomic annotation outputs were filtered in MG-RAST to only include those from Bacteria and Archaea domains. Viruses and eukaryotes were not used in the analysis because they were underrepresented in the metagenome. The functional gene characterization was obtained from the SEED platform [79], similar to previous analysis [24, 25]. Metagenomes were compared using proportional abundance, which is preferred to rarefaction [10, 68, 83].

### Quantitative Evaluation of Leopard Shark Microbiomes

To identify whether the taxonomy of the *T. semifasciata* epidermal microbiome remained consistent or varied over time, a PERMANOVA on the relative abundance of each taxon level (from phyla to genera) was conducted across years using the entire dataset [2, 83]. PERMANOVA is designed for non-parametric data, particularly when there is a larger number of variables compared with samples, unequal group sample size, and where there is no requirement for multivariate normalization because normalization is met by the permutation function [2, 3]. All data were fourth root transformed [55], which balances the effects of a community structured on a few abundant species and a community structured on all species, and thereby influenced by the occurrence of the rarest taxa [16, 17]. A similarity of percentages (SIMPER) analysis was used to identify the taxa contributing to the dissimilarity between years [14]. A principal coordinate analysis was performed on the taxonomic data to visualize the variation in the structure of the microbiomes over the years using a Bray–Curtis similarity, 100 = similar, 0 = no overlap. Last, a PERMDISP analysis compared the distribution of microbial taxa across each year around the year’s group centroid [4, 15]. These analyses were conducted initially on the entire dataset and then on the recurrent taxa.

We have identified two groups of microbial genera, including recurrent microbes (or core) defined as those genera that were present on every individual shark, on a presence/absence basis and non-recurrent microbes, defined as all other genera that were occasional members of the microbiome. The 19 most abundant recurrent (selected because they had a mean of > 1% of sequences across all metagenomes) and non-recurrent microbial taxa (abundances calculated within the recurrent/non-recurrent datasets, separately) were compared with a Bray–Curtis similarity analysis to identified groups of genera that had a similar proportional abundance across years, visualized with a dendrogram. A Pearson correlation was conducted to identify positive or negative relationships between these taxonomic groupings, displayed as a heatmap. The output of these two analyses was combined to show how the microbial genera related to one another. The analysis identified negative and positive correlations between the microbial groups found in the *T. semifasciata* shark epidermal microbiome.

We used metagenomics to describe the abundance of genes found by microbiome as a proxy for gene expression: although metagenomics does not measure which functional genes are being expressed at the point the sample was taken, it measures which functional genes are important for the bacteria in that environment [18, 24, 25]. There is a high level of correlation between the metagenomes and metatranscriptomes [34], where the abundance of a gene in metagenomes is a predictor of its expression level in the metatranscriptome and areas where the two analyses vary are associated with short-term changes in expression rather than bacterial functions that are under strong selective pressure and are well adapted to their environment [35, 36, 65]. For the leopard shark analysis, we used metagenomes to provide a functional potential, because of the stability of sampling DNA compared with mRNA in the marine setting. To identify whether the functional potential of the *T. semifasciata* epidermal microbiome remains stable or varies temporally, a PERMANOVA was conducted to compare variation in functional components across years. The SEED subsystem provides a hierarchal description in four levels including level 1 — major metabolic processes (such as carbohydrate metabolism); level 2 — metabolic pathways (such as di- and oligosaccharides); level 3 — gene clusters (such as 2-O-alpha-mannosyl-D-glycerate utilization); and level 4 — gene functions (such as mannosyl-D-glycerate utilization repressor MngR) [79] and all levels were tested. The SIMPER analysis identified potential functions that contributed to the dissimilarity between years. A principal coordinate analysis was performed to visualize the variation in the functional potential of the microbiomes over the years and a PERMDISP analysis compared the distribution of functional potential of *T. semifasciata* epidermal microbiome across years. All analyses were performed in PRIMER Version 6.1.15 and PERMANOVA ± Version 1.0.5 from PRIMER-E (2012). GraphPad Prism Version 8.3.1 was used to visualize the data.

We identified the functional potential of the 17 most proportionally abundant genera using the Krona plug-in [78] in MG-RAST [5, 69]. Only 17 genera were reported, because they occurred in sufficient abundance to explore the functions of the genera. The sequences were further investigated using Kyoto Encyclopedia of Genes and Genomes (KEGG) Orthology (KO) database’s KEGG mapper plug-in on MG-RAST [48, 49], which provided identification of metabolic and biochemical pathways. The proportion of sequences within each genus for each functional group was calculated as the number of sequences within a genus divided by the total number of sequences within the function across the 17 genera. The proportion of sequences associated with each function in each genus was categorized into one of four quartiles: > 75%, 50–75%, 25–50%, 1–25%, and 0% and visualized using a heatmap. The heatmap was used to visualize the connection between the microbes and the sharks over time and identified whether there was switching of specific functional roles between genera in the microbiome.

## Results

### Temporal Taxonomy of the Epidermal Microbiome

The epidermal microbiomes were constructed from 18 T*. semifasciata*, including seven individuals in 2013, three in 2015, and eight in 2017 caught in La Jolla, CA. Metagenomes ranged from 312,135 to 2,347,886 sequences that had 89,080,935–605,042,370 base pairs (Table 1). Of those sequences identified as protein coding, 24.0–83.7% were classified as unknown sequences, which could not be matched to the database (Table 1). The abundant phyla represented in the *T. semifasciata* epidermal microbiome were Proteobacteria (78.5 ± 2.0 S.E. %), Bacteroidetes (15.8 ± 2.3%), and Actinobacteria (2.1 ± 0.3%). Within the Proteobacteria, the classes Gammaproteobacteria (39.0 ± 2.3%) and Alphaproteobacteria (31.3 ± 2.2%) were evenly represented, with Betaproteobacteria (7.2 ± 0.8%) and Deltaproteobacteria (0.8 ± 0.2%) in lower relative abundance (SI Fig. 1). Within the Bacteroidetes, the Flavobacteria class made up 12.1% (± 2.2) of the metagenome and Actinobacteria class accounted for 2.4% (± 0.3) (SI Fig. 2).

**Table 1:** The *Triakis semifasciata* metagenome statistics, including collection date, total sequences, and proportion of the metagenome that was annotated by the database

Metagenome name	Date	Location	SRA Accession	Number of sequences	Annotated proteins (%)
LS 2 2013	2013	La Jolla	SAMN25656875	1060680	24.36
LS 3 2013	2013	La Jolla	SAMN25656876	1024296	20.16
LS 4 2013	2013	La Jolla	SAMN25656877	1154296	21.33
LS 5 2013	2013	La Jolla	SAMN25656878	2347886	19.79
LS 6 2013	2013	La Jolla	SAMN25656879	3346424	18.84
LS 7 2013	2013	La Jolla	SAMN25656880	2330953	20.36
LS 1 2015	2015	La Jolla	SAMN25656881	1143301	22.68
LS 2 2015	2015	La Jolla	SAMN25656882	2012275	15.87
LS 3 2015	2015	La Jolla	SAMN25656883	1635251	20.4
LS 1 2017	2017	La Jolla	SAMN25656884	701418	75.63
LS 2 2017	2017	La Jolla	SAMN25656885	898767	74.86
LS 3 2017	2017	La Jolla	SAMN25656886	636435	46.71
LS 4 2017	2017	La Jolla	SAMN25656887	324102	68.57
LS 5 2017	2017	La Jolla	SAMN25656888	312135	61.76
LS 6 2017	2017	La Jolla	SAMN25656889	693318	43.43
LS 7 2017	2017	La Jolla	SAMN25656890	613946	62.52
LS 8 2017	2017	La Jolla	SAMN25656891	1189453	43.69

**Fig. 1:**
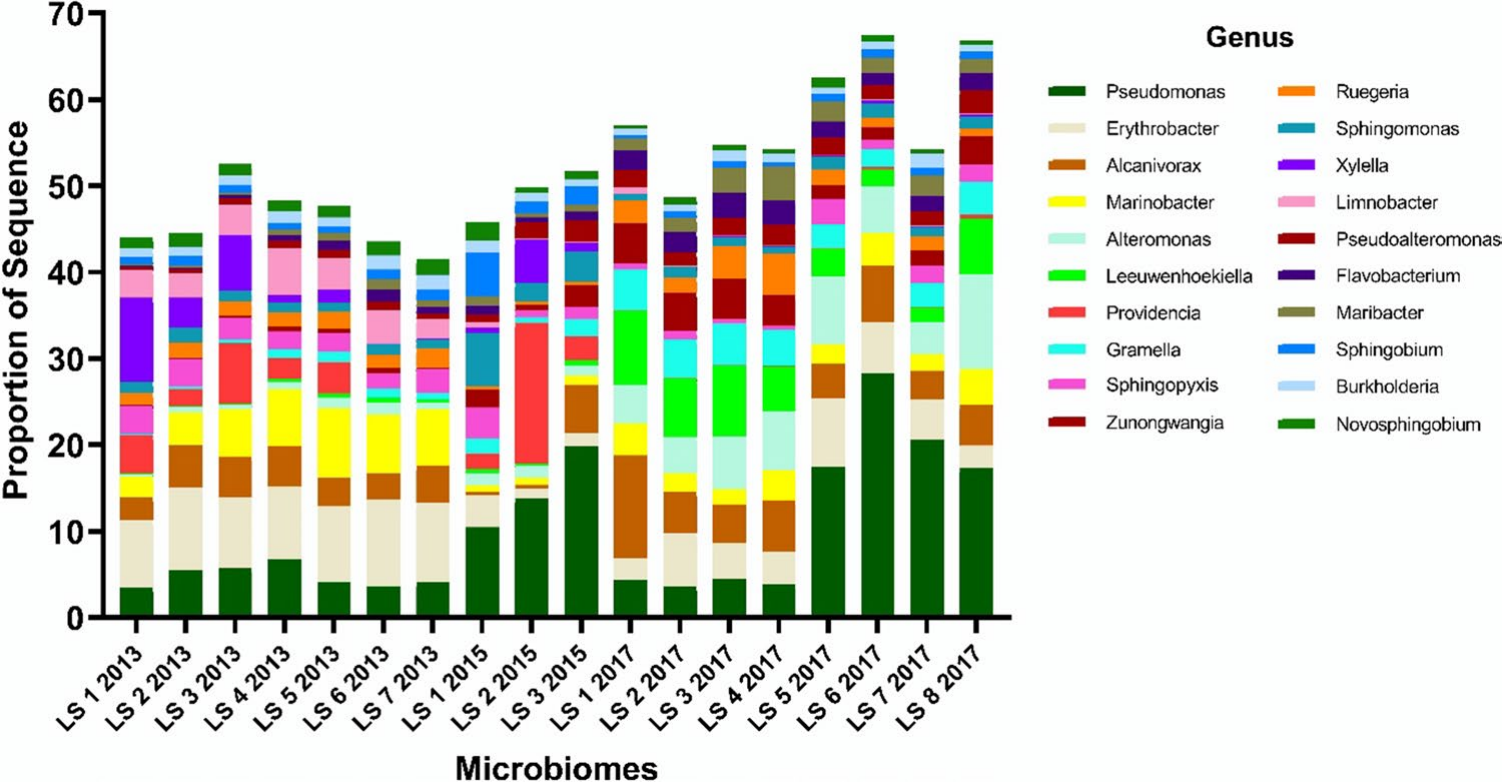
The relative proportions of the most abundant microbial genera represented in the *Triakis semifasciata* microbiome across 4 years

**Fig. 2:**
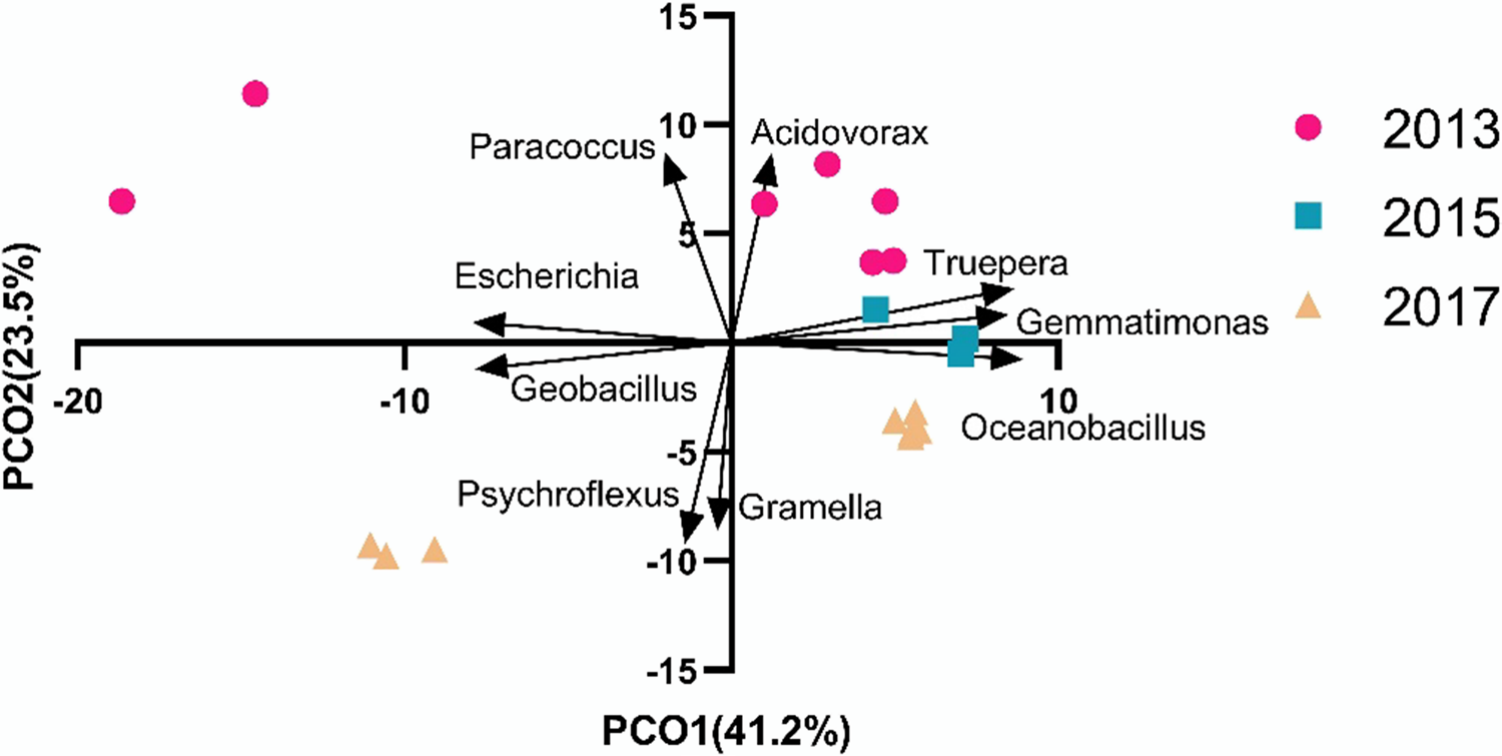
The microbial genera of the *Triakis semifasciata* epidermal microbiome clustered by year, analysed on proportional sequence distribution of all genera

Across the 18 leopard sharks, 597 genera made up the epidermal microbiome. Highly represented genera present within the microbiomes included *Pseudomonas* (mean 9.9 ± 1.8 S. E.%), *Erythrobacter* (5.9 ± 0.79%), *Leeuwenhoekiella* (2.5 ± 0.7%), and *Limnobacter* (1.5 ± 0.4%) (Fig. 1). The *T. semifasciata* microbiomes collected across the three time-points showed high taxonomic similarity between all individuals, with average Bray–Curtis similarity of 82.7 (0 = no overlap, 100 = total overlap) among all pairs of samples. The similarity of the genera on the sharks across time-points suggests a selective process is occurring and that the shark epidermis has a specialized microbiome (Fig. 2). The PERMANOVA analysis identified significant differences in the proportional abundances of all taxonomic levels between years (PERMANOVA: phylum, pseudo-*F*_df = 2_ = 3.696, *P*(perm) = 0.001; class, pseudo-*F*_df = 2_ = 3.628, *P*(perm) = 0.001; order, pseudo-*F*_df = 2_ = 3.222, *P*(perm) < 0.01; family, pseudo-*F*_df = 2_ = 2.762, *P*(perm) < 0.01; genus, pseudo-*F*_df = 2_ = 2.967, *P*(perm) = 0.049). The microbial genera of the *T. semifasciata* epidermal microbiomes formed distinct clusters for each year in the principal coordinate analysis, which explained 64.7% of the variation in the first two axes (Fig. 2). A few outliers identified in 2013 and 2017 may be associated with variation in an individual shark’s physiology, i.e., such as pregnancy.

The SIMPER analysis identified a similarity coefficient of 83.2 between 2013 and 2015, 80.4 for 2013 and 2017, and 84.6 for 2015 and 2017 (Bray–Curtis similarity index, 100 = similar, 0 = no overlap). Genera of the Gammaproteobacteria class contributed the most to the dissimilarity between the years, followed by genera in the Alphaproteobacteria and Betaproteobacteria, and the Flavobacteria class from the Bacteroidetes phylum (Table 2). The variation in the relative abundance of *Alcanivorax* and *Alteromonas* contributed the most to the dissimilarity across years.

**Table 2:** The genera that contributed to the flexibility in the *Triakis semifasciata* epidermal microbiome across 4 years, calculated by SIMPER analysis, with dissimilarity coefficient of 16.95 between 2013 and 2015, 19.71 for 2013 and 2017, and 15.32 for 2015 and 2017

Years compared	Phyla	Class	Genus	Percent contribution
2013 vs 2015	Proteobacteria	Gammaproteobateria	*Providencia*	0.9
			*Endorifia*	0.9
			*Marinobacter*	0.7
			*Pseudomonas*	0.6
			*Xylella*	0.6
			*Alcanivorax*	0.6
		Betaproteobacteria	*Liminobacter*	0.9
		Alphaproteobacteria	*Erythrobacter*	0.7
			*Hyphomonas*	0.6
			*Oceanibulbus*	0.7
2013 vs 2017	Bacteroidetes	Flavobacteria	*Leeuwenkoekiella*	0.9
			*Zuongwangia*	0.7
			*Gramella*	0.6
	Proteobacteria	Betaproteobacteria	*Limnobacter*	0.9
		Gammaproteobateria	*Providencia*	0.9
			*Alteromonas*	0.7
			*Xylella*	0.7
			*Endorifia*	0.5
		Alphaproteobacteria	*Hyphomonas*	0.5
		Planctomycetia	*Planctomyces*	0.5
2015 vs 2017	Proteobacteria	Gammaproteobateria	*Providencia*	1.4
			*Endorifia*	1.1
			*Psychrobacter*	0.9
			*Xylella*	0.8
			*Alcanivorax*	0.7
			*Altermonas*	0.7
			*Pseudomonas*	0.5
		Alphaproteobacteria	*Ruegeria*	0.6
		Betaproteobacteria	*Achromobacter*	0.6
	Bacteroidetes	Flavobacteria	*Leeuwenkoekiella*	1.0

Last, we explored the variance within years of microbial taxonomy with PERMDISP analysis. Each year the microbiome showed a similar amount of variation at each taxonomic level (PERMDISP: phylum, *F*_df = 2, 15_ = 2.120, *P*(perm) > 0.1; class, *F*_df = 2, 15_ = 2.439, *P*(perm) > 0.1; order, *F*_df = 2, 15_ = 1.513, *P*(perm) > 0.5; family, *F*_df = 2, 15_ = 2.019, *P*(perm) > 0.1; genus, *F*_df = 2, 15_ = 2.142, *P*(perm) > 0.05). The microbiomes from 2013, 2015, and 2017 had dispersion scores of 11.7 (± 1.8), 6.0 (± 0.8), and 10.1 (± 1.3) respectively. Therefore, the individual sharks had consistent taxon across the years; however, those taxa had proportional abundances that were flexible.

### Temporally Recurrent Microbes

**Fig. 3:**
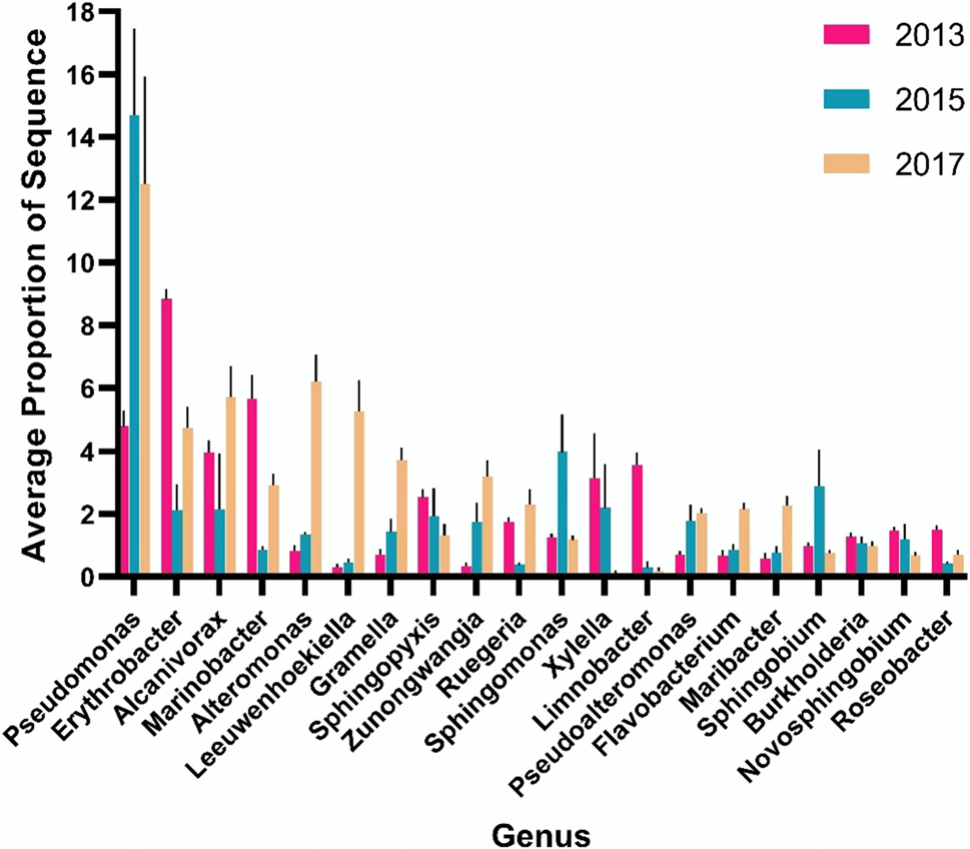
The mean relative abundance (± S.E.) of the 19 most abundant recurrent genera per year on the *Triakis semifasciata* epidermal microbiome, showing flexibility over the 4 years

Microbial genera present in all 18 metagenomes were categorized as “recurrent” which included 188 of 597 genera. From the 188 recurrent genera, we determined how stable the relative abundance was across the three sampled time-points and identified a significant difference (PERMANOVA: pseudo-*F*_df = 2, 15_ = 10.462, *P*(perm) = 0.001). The variation in the 19 most relatively abundant recurrent genera is shown in Fig. 3. For instance, the relative abundance of *Pseudomonas* was low in 2013; however, it was the most relative abundance genera in both 2015 and 2017. Conversely, *Erythrobacter* had the highest relative abundance in 2013, but lower relative abundance in both 2015 and 2017. We then determined if the same degree of variability persisted within the 188 recurrent microbes. The variability in the recurrent microbial genera, measured by PERMDISP (PERMDISP: *F*_df = 2_, _15_ = 0.029, *P*(perm) = 0.983), indicated no difference across years with 2013, 2015, and 2017 showing a dispersion of 4.1 (± 0.5), 4.1 (± 0.6), and 4.0 (± 0.2), respectively. Therefore, collectively the fluctuations in the dominate genera along with similar variability across time suggest flexible but regulated recurrent groups of microbes in the *T. semifasciata* epidermal microbiome.

### Relationship Between Recurrent and Non-recurrent Genera

**Fig. 4:**
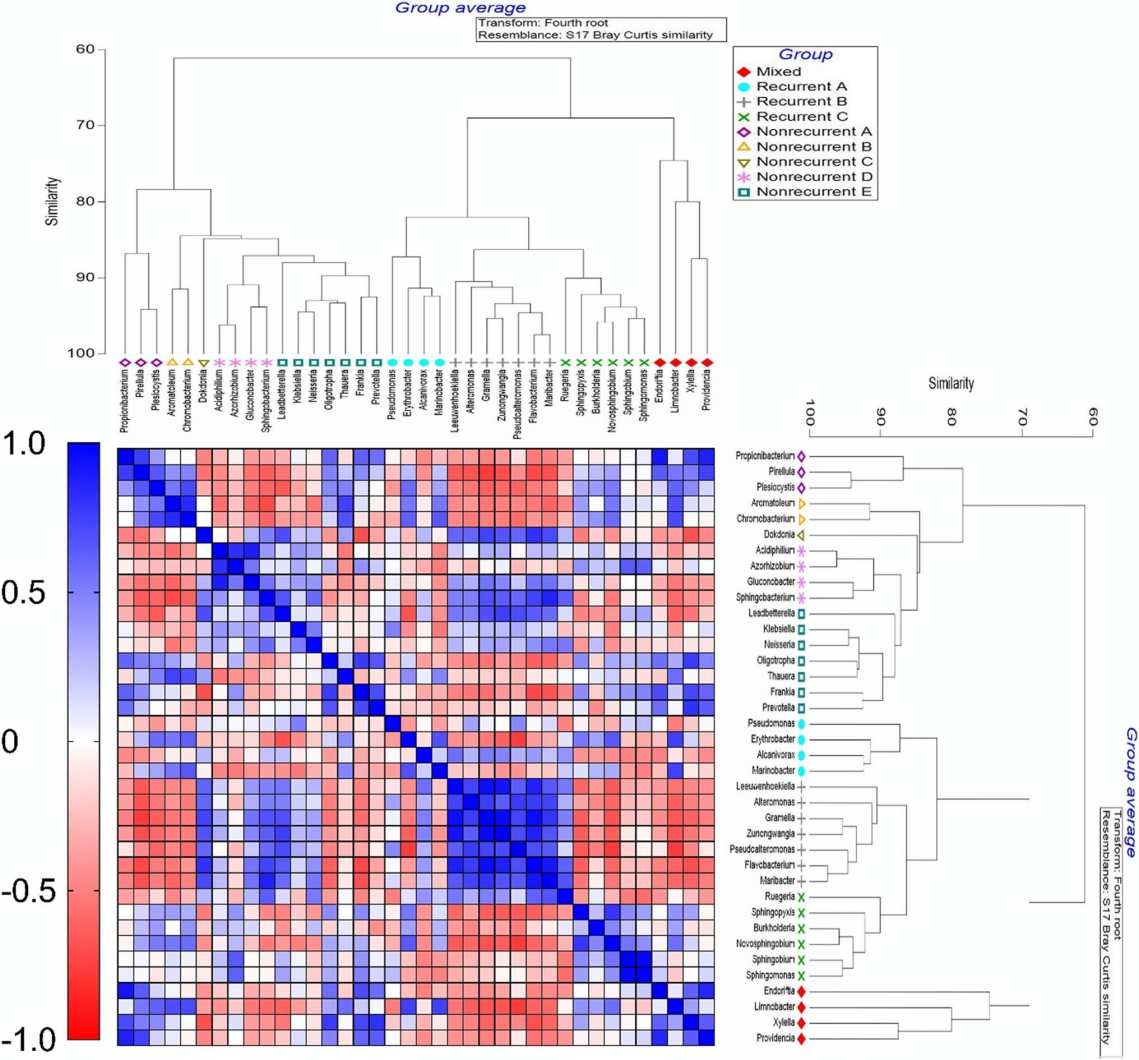
Positive and negative correlations occurred between the most abundant 19 recurrent and non-recurrent microbes in the *Triakis semifasciata* epidermal microbiome. Bray–Curtis similarity was used to cluster the genera with similar proportional abundance across years and the relationship with other genera was compared using Pearson’s correlation, displayed as a heatmap where red is a negative correlation and blue is a positive correlation

To investigate the relationship between the recurrent and non-recurrent genera (top 19 most relatively abundant genera not occurring across all sampling points), we first clustered the genera together with respect to their relative abundance across time-points based on Bray–Curtis similarity. Clustering identified three groups from the 19 dominant recurrent microbes labeled A, B, and C and five groups of the 19 most relative abundance non-recurrent microbes labeled A, B, C, D, and E (Fig. 4). The recurrent group A consisted of *Pseudomonas, Erythrobacter, Alcanivoriax, and Marinobacter*, which showed an even proportional abundance across the three time-points except *Erythrobacter and Marinobacter* which had higher proportional abundance in 2013. The recurrent group B consisted of *Leeuwenhoekiella, Altermonas, Gramella, Zunongwangia, Pseudoaltermonas, Flabacterium, and Maribacter* which had higher proportional abundance in 2017. The recurrent group C consisted of six genera including *Burkolderia, Novoshingobium, and Sphingopyxis* which had even distribution across the years; *Rugeria*, which had proportional higher abundance in 2013 and 2017; and *Sphingobium and Sphingomonas*, which had higher proportional abundance in 2015. The non-recurrent genera in group A included *Propionibacterium, Pirellula, and Plesiocystis and had* lower proportional abundance in 2017. The non-recurrent microbial group B consisted of *Aromatoleum and Chromobacterium* and had low but even proportional abundance across all years. The non-recurrent microbial group C only consisted of *Dokdonia*, which displayed higher proportional abundance in 2017. The non-recurrent group D included *Acidiphillium, Azorhizobium*, which had low but even proportional abundance across years, and *Gluconobacter and Sphingobacterium* which showed higher proportional abundance in 2017. The non-recurrent microbial group E, including *Leadbetterella* with highest proportional abundance in 2015 and *Klebsiella, Neisseria, and Thauara* with even proportional abundance and *Oligotropha, Frankia, and Leadbetterella* displayed its highest proportional abundance in 2015. The last group included recurrent *Xylella and Limnobacter* and non-recurrent *Endoritia and Providencia* genera, therefore forming the mixed cluster on the dendrogram (Fig. 4).

We then investigated the correlative relationship of recurrent and non-recurrent groups (Fig. 4). The heatmap showed that *Erythrobacter* and *Marinobacter* (recurrent group A) were negatively correlated with all microbes in recurrent group B. Whereas *Pseudomonas* and *Alcanivorax* (recurrent group A) showed positive correlations with recurrent group B. Recurrent group A, however, was positivity correlated with non-recurrent groups A and B. In contrast, recurrent group B was negatively correlated with non-recurrent groups A and B but strongly associated with non-recurrent group C, *Dokdonia*. Recurrent group C was positively correlated with non-recurrent groups A, B, D, and E and the mixed cluster (Fig. 4). These associations between taxa will be investigated further in the functional analysis to identify metabolic processes that may drive the microbiome composition.

### Functional Potential of the Shark Epidermal Microbiome

**Fig. 5:**
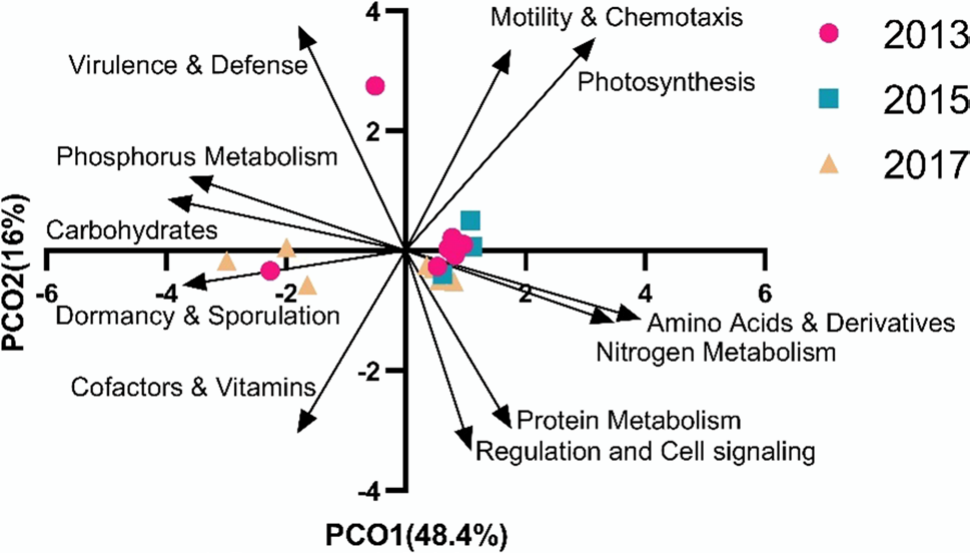
The functional potential of the *Triakis semifasciata* epidermal microbiome was stable across 4 years, shown using a principal component analysis

Annotated microbial sequences were categorized into 26 major metabolic processes (SEED level 1 subsystems), 153 metabolic pathways (level 2), 949 functional gene clusters (level 3), and 5,358 functional genes (level 4). The functional profiles were highly stable across the three time-points as evidenced by the high functional similarity between individual sharks (mean 97.7, Bray–Curtis similarity index). There was no significant difference between time-points in the functional potential of the microbiome across the three hierarchical levels (PERMANOVA: major metabolic processes, pseudo-*F*_df = 2, 15_ = 1.8, *P*(perm) > 0.05; metabolic pathways, pseudo-*F*_df = 2, 15_ = 0.525, *P*(perm) > 0.5; functional gene clusters, pseudo-*F*_df = 2, 15_ = 1.182, *P*(perm) > 0.1). The similarity of the level 1 functions across time-points was shown by the single dense cluster on the principal coordinate analysis that explained 64.4% of the variation in the first two axes (Fig. 5).

The major metabolic processes (SEED level 1) in the leopard shark microbiome included carbohydrate metabolism (mean 11.0 ± 0.3%); amino acids and derivatives (9.2 ± 0.2%); protein metabolism (7.9 ± 0.1%); and fatty acids, lipids, and isoprenoids (2.6 ± 0.1%) (SI Fig. 3). We also looked at the major metabolic processes with lower abundance within our metagenomes because these functions are expected to provide microbes with the ability to fill unique ecological niches within the microbiome. Within the major metabolic process; nitrogen metabolism (1.2 ± 0.1%), there were 11 functional gene clusters (level 3) in the microbiome including ammonia assimilation (0.5 ± 0.01%), nitrate and nitrite ammonification (0.38 ± 0.001%), and denitrification (0.1 ± 0.02%), but relatively few sequences were associated with nitrogen-fixing genes (0.02 ± 0.007%) suggesting high levels of organic nitrogen that are present on the shark. Urea decomposition (0.3 ± 0.02%) was present and there were a few sequences associated with trimethylamine N-oxide (TMAO) reductase. Within the fatty acid biosynthesis and metabolism, major metabolic processes were fatty acid biosynthesis FASII (0.7 ± 0.02%), fatty acid degradation regulons (0.2 ± 0.02%), and fatty acid metabolism (0.13 ± 0.01%). The functional gene clusters that complete protein biosynthesis and degradation are ATP-dependent proteolysis in bacteria (0.4 ± 0.01%), bacterial proteasome (0.3 ± 0.02%), protein degradation (0.2 ± 0.01%), nucleolar protein complex (1.0 ± 0.15%), aminopeptidase (0.18 ± 0.01%), metallocarboxypeptidase (0.04 ± 0.006%), and bacterial ribosome LSU (0.4 ± 0.02%). A high proportional abundance of flagellar motility (1.3 ± 0.1%) and lower proportional abundance of bacterial chemotaxis (0.4 ± 0.02%) are consistent with low biofilm formation predicted on the shark epidermis. There was a high proportional abundance of genes associated with resistance to antibiotics and toxic compounds (5.18 ± 0.1%), including genes associated with cobalt-zinc-cadmium resistance, multidrug resistance efflux pumps, and copper homeostasis, which are used in heavy metal detoxification, consistent with bioaccumulation of heavy metals and toxins by the large-bodied sharks.

### Microbial Gene Function Across Genera

**Fig. 6:**
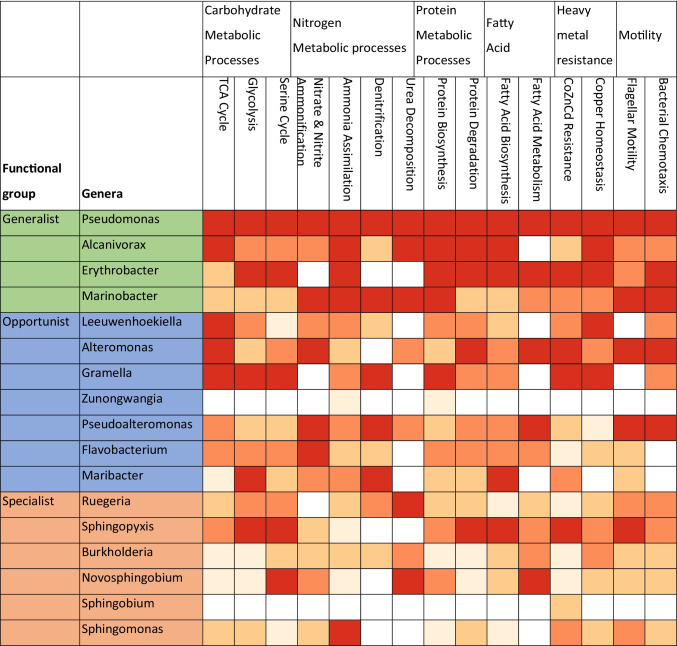
Heatmap of the mean proportion of genes associated with each metabolism in the 17 most abundant recurrent taxon in group A (aqua), group B (gray), and group C (green) shows genes were mostly shared across taxon, but some specialization occurred. The proportion of sequences were divided into quartiles, where darker colors represent higher percentile (> 75 dark red, 50–75 orange/red, 25–50 orange, and 0–25 pale orange). No color represented no sequences for that metabolic process held by that genus

The 17 dominant recurrent genera were used to investigate whether functional genes were equally dispersed across the genera or confined to a small subset of microbes (Fig. 6). *Pseudomonas* had the highest proportion of sequences annotated to all metabolic processes, including those involved in nitrate and nitrite ammonification and denitrification. *Pseudomonas* and *Alcanivorax* also conducted ammonia assimilation. These two genera were positively correlated (Fig. 6) with most other recurrent genera, suggesting nitrogen metabolism within this group may facilitate survival of other *T. semifasciata* epidermal microbiome members. In comparison, *Marinobacter* also specialized in these two functions plus urea decomposition and was negatively correlated *Pseudomonas* and *Alcanivorax*. *Ruegeria* and *Novosphingobium* were the only other genera that had a high proportional abundance of genes associated with urea decomposition, which may have caused the negative correlation with recurrent group B. The genera within recurrent group B had a high proportion of genes associated with carbon metabolism, with *Gramella* having a high proportion of genes within TCA cycle, glycolysis and serine cycle. *Maribacter* has a high proportion of genes in glycolysis, denitrification, and fatty acid biosynthesis. Many genera, including *Pseudomonas*, *Alcanivorax*, *Erythrobacter*, *Leeuwenhoekiella*, *Altermonas*, *Gramella*, and *Flavobacterium*, had protein biosynthesis and degradation, and fatty acid biosynthesis genes, which are associated with the shark ketosis dominated metabolism. Genera within recurrent group C, including *Ruegeria*, *Sphingopyxis*, and *Novosphingobium*, had a high proportion of genes associated with denitrification and fatty acid biosynthesis. All 17 genera, except one, had sequences that matched to copper, cobalt, and cadmium resistance and copper homoeostasis genes, suggesting the importance of heavy metal tolerance or manipulation for microbes living on the surface of sharks.

## Discussion

The *T. semifasciata* epidermal microbiome was consistent between individual sharks with many genera shared across the three time-points spanning 4 years, one of the longest studies of a marine vertebrate microbiome to date. A recurrent set of 188 microbial genera were present on all individuals. However, there was flexibility in the proportional makeup of the microbiome genera across years, but the microbiome variability was consistent across years, suggesting similar ecological dynamics are occurring from year to year, despite some switching in dominate microbial genera. Regardless of the proportional abundance of each microbial genera, the functional potential of the microbiome was constant, suggesting that the epidermal microbes were responding to the unique environment of the *T. semifasciata* epidermis and that functional redundancy across microbial genera may allow the proportional switching of the genera observed across time-points.

### Leopard Shark Microbiome Exhibits Specificity and Flexibility

The *T. semifasciata* epidermal microbiome has a high relative abundance of *Pseudomonas*, *Erythrobacter*, *Alcanivorax*, and *Marinobacter*; whereas the thresher shark (*Alopias vulpinus*) epidermal microbiome had a high relative abundance of *Pseudoalteromonas*, *Erythrobacter*, *Idiomarina*, and *Limnobacter* [26], and the Black-tip reef sharks (*Carcharhinus melanopterus*) epidermal microbiome a high relative abundance of *Psychrobacter*, *Pseudoalteromonas*, *Rhodobacter*, and *Alteromonas* [82]. While some genera were shared across shark species, the proportional abundance differed, suggesting that each elasmobranch microbiome has distinct characteristics which selects for differing microbiome members.

The highly shared microbiome across individual sharks within a time-point and flexible communities across time-points coupled with evidence that shark species microbiomes are distinct may be caused by the dermal denticles [13, 23] and metabolites secreted into the skin mucus as a result of the sharks metabolism. Dermal denticles alter the hydrodynamic properties of water close to the epidermis, reducing drag on the shark [91] and micro-organism growth [99, 103]. A study that tested the recruitment of two lab microbial species found that only one species was able to successfully recruit, and biofilms did not develop [13, 50]. These features are consistent across the microbiome of four shark species [26, 28]. The epidermal microbiome of embryonic skates showed a dramatic change at the time of dermal denticle hardening, going from similar across all body parts to a unique microbiome on the epidermis [72], confirming the effect of the dermal denticles in microbiome structure.

Modelling of microbial community dynamics on surfaces with high structural complexity, like the conditions on a shark, suggests there is lower interspecific competition that promotes limited extinction, and results in a chaotic spatial distribution pattern of microbial species [61]. An expected outcome in this situation is a high level of shared microbes across individuals, but with varying abundance patterns. The *T. semifasciata* epidermal microbiome supports this expectation where 188 genera coexisted on all sharks across the three time-points. The flexibility in the relative abundance of those genera suggests that the epidermis creates multiple niches driven by the physical structure of the surface. Similarly, the skin microbiome of three lizard species was more diverse and had a larger core number of OTUs than that of six frog species, which may be associated with the structured nature of the rough surface created by the lizard species skin relative to the mucus covered frog epidermis and lower levels of antimicrobial peptides on the lizard skin [98]. The high diversity of the *T. semifasciata* microbiome may be further enhanced through potential interactions among groups of co-occurrence genera which drive niche partitioning and resource sharing (Fig. 4). For example, *Pseudomonas* and *Alcanivorax* showed positive correlations with recurrent group B members, including *Leeuwenhoekiella*, *Altermonas*, *Pseudoaltermonas*, *Flabacterium*, and *Maribacter*, suggesting that these two genera facilitate one another in some capacity through shared resources. Conversely, *Erythrobacter* and *Marinobacter* were negatively correlated with all microbes in recurrent group B, suggesting strong competition; this group was however positively correlated with non-recurrent groups A and B, suggesting transient microbes add to the microbiome’s growth at different years.

### Functions for Life on a Shark Skin

We investigated the gene functions present in the groups of microbial genera that may explain the positive or negative relationships between genera in Fig. 4. In addition, because host metabolism and microbial functions are linked [59, 62], we identified functional genes in each genus that may be linked to the host metabolism and classify three broad functional groups, including generalists (recurrent group A), opportunists (recurrent group B) and specialists (recurrent group C) based on the presence of potential genes in these microbial genera. The group A recurrent microbes are typical marine species that have highly flexible genomes and growth strategies, including heterotrophic and photo-heterotrophic growth. These organisms can utilize many of the nutrients anticipated to be present on the shark epidermis or excreted in the mucus, including proteins, fatty acids, and disulfides [70]. *Pseudomonas*, for example, is capable of mixotrophy [38] and the positive correlation of *Pseudomonas* with many other microbes (Fig. 4) may have been enhanced by traits, such as phosphate solubilization, and ammonia production [37, 102].

*Alcanivorax* species, a highly abundant recurrent group A microbe, has the genes for reducing alkane hydrocarbons, and possessed many enzymes for β-oxidation of fatty acids, the glyoxylate bypass, andgluconeogenesis. These gene functions carried by *Alcanivorax* suggest they break down the complex hydrocarbons and fatty acid–rich metabolites and may facilitate other member of the microbiome by providing simpler metabolic products that can be utilized. They also include enzymes for synthesis of riboflavin and unsaturated fatty acids and cardiolipin [84]. Cardiolipin is a diphosphatidylglycerol lipid [85] and is more highly saturated in sharks than other vertebrates [87].

*Erythrobacter* species, a highly abundance group A microbe that was negatively correlated with many other microbes, are mostly aerobic anoxygenic phototrophic bacteria, containing bacteriochlorophyll a, but lack the genes of autotrophic CO_2_ fixation pathway, thus photoheterotrophic metabolism requiring a supply of organic substrates. Several *Erythrobacter* species have genes encoding enzymes for glycolysis and the tricarboxylic acid cycle but lack genes for nitrogenase or nitrate reductase thus are reliant on other forms of organic nitrogen [52]. *Erythrobacter* carry heavy metal resistance genes, including resistance to lead, cadmium, zinc, mercury, nickel, cobalt, and arsenicals [104]. The behavior of the leopard sharks, being in shallow water with higher light conditions, may have provided the *Erythrobacter* with a competitive advantage over other microbial groups.

The last group A recurrent microbe was *Marinobacter* and these organisms are opportunistic generalists that switch rapidly between lithoheterotrophy to heterotrophy, in both anaerobic and aerobic conditions, in response to nutrient pulses [40]. *Marinabacter* can respire inorganic compounds that are usually found in metal rich environments [40], and we show they have many genes associated with metal resistance and transport.

The recurrent group B microbes, the opportunists, were negatively correlated with most other groups and had a high relative abundance of genes associated with carbohydrate metabolic processes. *Gramella* species degrades high molecular weight organic matter and encoding hydrolytic enzymes predicting a preference for polymeric carbon sources and have range of gliding motility genes that provide for surface adhesion [7], features that may enable the organism to live on the surface of elasmobranchs. However, these microbes had fewer genes in ammonia assimilation, promoting their coexistence with the more abundant species. *Leeuwenhoekiella*, a marine Flavobacteria, has heterotrophic growth, is pigmented, and often found in association with photosynthetic microbes [94], such as those described in the recurrent group A genera. *Alteromonas* are non-phototrophic, heterotrophic, with flexible genome for taking advantage of influx of nutrients, particularly polymers, and some degrade polycyclic aromatic hydrocarbons [66]. *Alteromonas* have high flexibility in the metal resistant genes, such as the copper-zinc-cobalt genes and efflux pumps [60] and these were highly abundant in this genus in the *T. semifasciata* microbiome. *Pseudoalteromonas* and *Alteromonas* utilized extracellular hydrolysis as the major decomposition pathway of peptides and released fragments of amino acids into the surrounding environment [57] and may have enabled them to outcompete *Marinobacter* which is unable to conduct extracellular hydrolysis processes [40]. However, these free amino acids may be used by other microbes, such as the non-recurrent group C and D microbes.

The group C recurrent microbes, the specialists, had low relative abundance overall but showed high relative abundance of a few metabolic pathways (Fig. 5). *Ruegeria* had a high proportion of sequences associated with urea metabolism, compared with its relatively low proportional abundance. *Ruegeria* are able to use trimethylamine (TMA) and trimethylamine N-oxide (TMAO) as an energy source to produce intracellular ATP [54]. TMAO and TMA are used by elasmobranchs for osmoregulation and TMAO-associated genes were identified in the microbiome. *Sphingopyxis* also had low relative abundance in the microbiome, utilizing carbon and nitrogen compounds as substrates for growth but are generally slow growing [74]. Serine, a pathway that was overrepresented in *Sphingopyxis*, can serve as a nitrogen source for the growth of some species, but not as a carbon source [100]. *Sphingomonas* are closely related to *Sphingoyxis* and both produce exopolysaccharides (EPS) [12]. The production of these EPS compounds may promote biofilm development on the shark skin. Therefore, each recurrent microbial group was providing a different set of functional genes that enhanced coexistence and reflected the products that may be associated with the shark mucus.

### Gene Functions Linked to Host Metabolic Processes

**Fig. 7:**
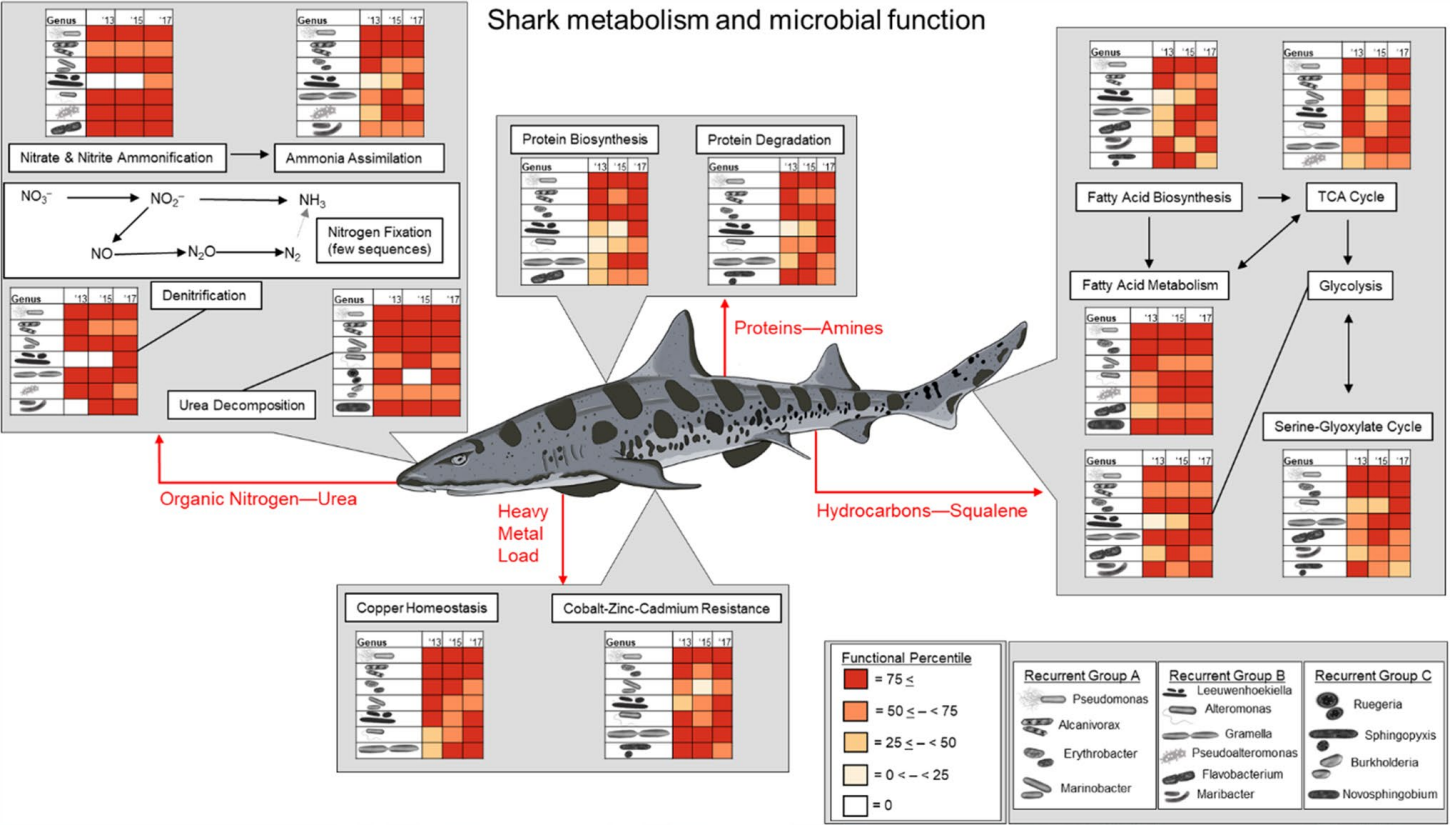
The links between the functional potential of the *Triakis semifasciata* epidermal microbiome and the host metabolism. The proportion of each gene in each taxon was identified and classed into quartiles. Most taxa had most functions suggesting the microbiome displays weak competition and is responding to the metabolic processes of the host

The stability of the functional potential of the epidermal microbiome suggests the elasmobranch physiology and dermal denticle topography may both be drivers of microbiome structure, and we have developed a model to link key microbiome functions with shark metabolism (Fig. 7). Elasmobranchs rely on protein as the primary energy source and use ketogenic pathways that produce high ketone bodies and hydrocarbons [88]. The functions that were present in metagenomes suggests that the microbes were responding to the presence of these compounds in the minimal mucus that covers the sharks. For example, *Alcanivorax* and *Marinobacter* carried the genes to degrade hydrocarbons. There are few descriptions of the compounds within the mucus of elasmobranchs, and these included high levels of proteins [95] with moderate to high disulfide concentrations [71]. A recent transcriptome analysis of the integument of cookie cutter sharks identified glyceraldehyde-3-phosphate dehydrogenase and fructose-bisphosphate aldolase A transcripts, both playing key roles in the glycolysis and gluconeogenesis [22] suggesting these products may be available to the microbial community. Protein metabolism along with nitrate and nitrite ammonification and urea decomposition with genes such as trimethylamine N-oxide (TMAO) reductase genes were identified in the microbiomes. The low levels of nitrogen fixation genes and the high proportional abundance of nitrogen metabolism and urea decomposition genes within the microbiome suggest the elasmobranch skin is a nitrogen-rich environment [29, 41]. Proteasomes, metallocarboxypeptidase, aminopeptidase genes, protein degradation, and biosynthesis functional genes suggest a propensity of the microbes for protein degradation. An experiment on a human skin modelled showed that mixed microbiome communities had a greater positive effect on the skin function, including the mediation of the epidermal layer thickness, reduction in the number of actively proliferating cells, and increased filaggrin expression [59], suggesting that there is a two-way interaction between the microbes and host metabolic processes. The epidermal microbiome of embryonic skates showed a dramatic change at the time of dermal denticle hardening, going from similar to other body parts prior to denticle formation to unique post denticle formation [72], supporting our hypothesis that the dermal denticles affect microbiome structure. There was no evidence of dentine, hydrolysate, or collagen degradation in the shark skin microbiome, suggesting that the microbes are not breaking down the dermal denticles, which signifies a lack of negative impact on the sharks, at least in the form of degrading the dermal denticles. Therefore, a commensal connection between *T. semifasciata* and the epidermis microbiome may be established through the passive resource subsidization of these microbiome functions.

Cobalt-zinc-cadmium resistance and ton/tol outer membrane transport system associated with bacterial toxins [93] are highly prevalent functional genes shared across epidermis microbiomes of *T. semifasciata* (this study), *A. vulpinus* [26], *Rhincodon typus*, and *Urobatis halleri* [28]. Elasmobranchs bioaccumulate heavy metals via their food and shunt these compounds to the epidermis [33, 67], and absorb heavy metals from the environment [45, 45, 46, 46]. The epidermal microbiomes are responding to the presence of these heavy metals and future studies should measure heavy metal concentration in the skin in conjunction with the microbiome. The prevalence of heavy metal resistance genes may provide a biomarker of elasmobranchs health and identify those exposed to anthropogenic sources of heavy metals in the environment, providing an early warning sign of declining health.

## Conclusions

The highly structured epidermis of elasmobranchs, in this case, *T. semifasciata*, promotes a diverse microbiome that has a flexible taxonomic makeup where many genera coexist. The microbial community was maintained by functional redundancy driven by the taxonomic flexibility and various microbes “flip-flopping” in abundance with one another, which kept the microbial niches filled across the years. The high relative abundance of genes involved in nitrification and denitrification, urea decomposition, and heavy metal resistance in the epidermis microbiome across all *T. semifasciata* individuals suggests a connection with the host metabolism potentially through passive subsidization of organic nitrogen, proteins, hydrocarbons, and heavy metals. Our results coupled with other host microbiomes studies, such as the skin surface microbiome of lizards, provide evidence that a structured surface is important for supporting and maintaining diverse skin surface microbiomes. Further efforts to understanding the effects of structure on driving skin microbiome communities will greatly enhance the ability to predict microbiome function of the largest organ in the vertebrate body.

## Supplementary Information

Below is the link to the electronic supplementary material.

Supplementary file1 (DOCX 504 KB)

## Data Availability

All data is publically available on the SRA database under BioProject PRJNA803589.

## References

[1] Albecker MA, Belden LK, McCoy MW (2019). Comparative analysis of anuran amphibian skin microbiomes across inland and coastal wetlands. Microb Ecol.

[2] Anderson MJ, Colton T, Balakrishnan N, Everitt B, Piegorsch W, Ruggeri F, Teugels J (2017). Permutational multivariate analysis of variance (PERMANOVA). Wiley statsref: statistics reference online.

[3] Anderson MJ, Walsh DCI, Clarke KR, Gorley RN, Guerra-Castro E (2017). Some solutions to the multivariate Behrens-Fisher problem for dissimilarity-based analyses. Aust N Z J Stat.

[4] Anderson MJ (2006). Distance-based tests for homogeneity of multivariate dispersions. Biometrics.

[5] Aziz RK, Bartels D, Best AA, DeJongh M, Disz T, Edwards RA, Formsma K, Gerdes S, Glass EM, Kubal M, Meyer F, Olsen GJ, Olson R, Osterman AL, Overbeek RA, McNeil LK, Paarmann D, Paczian T, Parrello B, Pusch GD, Reich C, Stevens R, Vassieva O, Vonstein V, Wilke A, Zagnitko O (2008) The RAST server: Rapid annotations using subsystems technology. BMC Genom 9:1–15 10.1186/1471-2164-9-75 PMC2265698 18261238

[6] Ballantyne JS (1997). Jaws: the inside story. The metabolism of elasmobranch fishes. Comp Biochem Physiol B: Biochem Mol Biol.

[7] Bauer M, Kube M, Teeling H, Richter M, Lombardot T, Allers E, Wurdemann CA, Quast C, Kuhl H, Knaust F, Woebken D, Bischof K, Mussmann M, Choudhuri JV, Meyer F, Reinhardt R, Amann RI, Glockner FO (2006). Whole genome analysis of the marine Bacteroidetes‘Gramella forsetii’ reveals adaptations to degradation of polymeric organic matter. Environ Microbiol.

[8] Bierlich KC, Miller C, DeForce E, Friedlaender AS, Johnston DW, Apprill A (2018) Temporal and regional variability in the skin microbiome of humpback whales along the Western Antarctic Peninsula. Appl Environ Microbiol 84 10.1128/AEM.02574-17 PMC5812929 29269499

[9] Caballero S, Galeano AM, Lozano JD, Vives M (2020). Description of the microbiota in epidermal mucus and skin of sharks (Ginglymostoma cirratum and Negaprion brevirostris) and one stingray (Hypanus americanus). Peerj.

[10] Calle ML (2019). Statistical analysis of metagenomics data. Genomics Inform.

[11] Cantu VA, Sadural J, Edwards RA (2019). PRINSEQ ++, a multi-threaded tool for fast and efficient quality control and preprocessing of sequencing datasets. PeerJ Preprints.

[12] Chang AP, Qian J, Li H, Wang YL, Lin JY, He QM, Shen YL, Zhu H (2021). 'Characterization and function of a novel welan gum lyase from marine Sphingomonas sp. WG. Front Microbiol.

[13] Chien HW, Chen XY, Tsai WP, Lee M (2020) Inhibition of biofilm formation by rough shark skin-patterned surfaces. Colloids Surf B-Biointerfaces 186 10.1016/j.colsurfb.2019.110738 31869602

[14] Clarke KR, Somerfield PJ, Gorley RN (2008). Testing of null hypotheses in exploratory community analyses: similarity profiles and biota-environment linkage. J Exp Mar Biol Ecol.

[15] Clarke KR, Somerfield P, Gorley RN (2016). Clustering in non-parametric multivariate analyses. J Exp Mar Biol Ecol.

[16] Clarke KR, Warwick RM (1994) Changes in marine communities: an approach to statistical analyses and interpretation. Natural Environment Research Council, Plymouth.

[17] Clarke KR, Gorley RN, Somerfield PJ, Warwick RM (2014) Changes in marine communities: An approach to statistical analysis and interpretation. 3nd Edition, PRIMER-E, Ltd., Phymouth Marine Laboratory, Plymouth.

[18] Coelho LP, Alves R, Del Rio AR, Myers PN, Cantalapiedra CP, Giner-Lamia J, Schmidt TS, Mende DR, Orakov A, Letunic I, Hildebrand F, Van Rossum T, Forslund SK, Khedkar S, Maistrenko OM, Pan S, Jia L, Ferretti P, Sunagawa S, Zhao XM, Nielsen HB, Huerta-Cepas J, Bork P (2022) Towards the biogeography of prokaryotic genes. Nature 601:252–256. 10.1038/s41586-021-04233-4 10.1038/s41586-021-04233-4 PMC7613196 34912116

[19] Compagno LJV, Dando M, Fowler SL (2005) Sharks of the world. Princeton, NJ: Princeton University Press

[20] Costello EK, Stagaman K, Dethlefsen L, Bohannan BJ, Relman DA (2012). The application of ecological theory toward an understanding of the human microbiome. Science.

[21] de Sousa Rangel B, Hammerschlag N, Sulikowski JA, Moreira RG (2021). Dietary and reproductive biomarkers in a generalist apex predator reveal differences in nutritional ecology across life stages. Mar Ecol Prog Ser.

[22] Delroisse J, Duchatelet L, Flammang P, Mallefet J (2021) Photophore distribution and enzymatic diversity within the photogenic integument of the cookie-cutter shark Isistius brasiliensis (Chondrichthyes: Dalatiidae). Front Mar Sci 8:1–11

[23] Dillon EM, Norris RD, O'Dea A (2017). Dermal denticles as a tool to reconstruct shark communities. Mar Ecol Prog Ser.

[24] Dinsdale EA, Edwards RA, Hall D, Angly F, Breitbart M, Brulc JM, Furlan M, Desnues C, Haynes M, Li LL, McDaniel L, Moran MA, Nelson KE, Nilsson C, Olson R, Paul J, Brito BR, Ruan YJ, Swan BK, Stevens R, Valentine DL, Thurber RV, Wegley L, White BA, Rohwer F (2008). Functional metagenomic profiling of nine biomes. Nature.

[25] Dinsdale EA, Pantos O, Smriga S, Edwards RA, Angly F, Wegley L, Hatay M, Hall D, Brown E, Haynes M, Krause L, Sala E, Sandin SA, Thurber RV, Willis BL, Azam F, Knowlton N, Rohwer F (2008). Microbial ecology of four coral atolls in the Northern Line Islands. Plos One.

[26] Doane MP, Haggerty JM, Kacev D, Papudeshi B, Dinsdale EA (2017). The skin microbiome of the common thresher shark (Alopias vulpinus) has low taxonomic and gene function B-diversity. Environ Microbiol Rep.

[27] Doane MP, Kacev D, Harrington S, Levi K, Pande D, Vega A, Dinsdale EA (2018). Mitochondrial recovery from shotgun metagenome sequencing enabling phylogenetic analysis of the common thresher shark (Alopias vulpinus). Meta Gene.

[28] Doane MP, Morris MM, Papudeshi B, Allen L, Pande D, Haggerty JM, Johri S, Turnlund AC, Peterson M, Kacev D, Nosal A, Ramirez D, Hovel K, Ledbetter J, Alker A, Avalos J, Baker K, Bhide S, Billings E, Byrum S, Clemens M, Demery AJ, Lima LFO, Gomez O, Gutierrez O, Hinton S, Kieu D, Kim A, Loaiza R, Martinez A, McGhee J, Nguyen K, Parlan S, Pham A, Price-Waldman R, Edwards RA, Dinsdale EA (2020) The skin microbiome of elasmobranchs follows phylosymbiosis, but in teleost fishes, the microbiomes converge. Microbiome 8:1–15 10.1186/s40168-020-00840-x PMC7293782 32534596

[29] Dowd WW, Harris BN, Cech JJ, Kultz D (2010). Proteomic and physiological responses of leopard sharks (Triakis semifasciata) to salinity change. J Exp Biol.

[30] Edwards RA, Haggerty JM, Cassman N, Busch JC, Aguinaldo K, Chinta S, Vaughn MH, Morey R, Harkins TT, Teiling C, Fredrikson K, Dinsdale EA (2013) Microbes, metagenomes and marine mammals: enabling the next generation of scientist to enter the genomic era. BMC Genomics 14. 10.1186/1471-2164-14-600 PMC3766688 24007365

[31] Egan S, Gardiner M (2016). Microbial dysbiosis: rethinking disease in marine ecosystems. Front Microbiol.

[32] Engel K, Sauer J, Junemann S, Winkler A, Wibberg D, Kalinowski J, Tauch A, Caspers BA (2018). Individual- and species-specific skin microbiomes in three different estrildid finch species revealed by 16S amplicon sequencing. Microb Ecol.

[33] Escobar-Sanchez O, Galvan-Magana F, Rosiles-Martinez R (2010). Mercury and selenium bioaccumulation in the smooth hammerhead shark, Sphyrna zygaena Linnaeus, from the Mexican Pacific Ocean. Bull Environ Contam Toxicol.

[34] Franzosa EA, Morgan XC, Segata N, Waldron L, Reyes J, Earl AM, Giannoukos G, Boylan MR, Ciulla D, Gevers D, Izard J, Garrett WS, Chan AT, Huttenhower C (2014). Relating the metatranscriptome and metagenome of the human gut. Proc Natl Acad Sci U S A.

[35] Gilbert JA, Field D, Huang Y, Edwards R, Li W, Gilna P, Joint I (2008). Detection of large numbers of novel sequences in the metatranscriptomes of complex marine microbial communities. Plos One.

[36] Gilbert JA, Meyer F, Schriml L, Joint IR, Muhling M, Field D (2010). Metagenomes and metatranscriptomes from the L4 long-term coastal monitoring station in the Western English Channel. Stand Genomic Sci.

[37] Goswami D, Vaghela H, Parmar S, Dhandhukia P, Thakker J (2013). Plant growth promoting potentials of Pseudomonas spp. strain OG isolated from marine water. J Plant Interact.

[38] Guo H, Chen C, Lee DJ (2019). Nitrogen and sulfur metabolisms of Pseudomonas sp. C27 under mixotrophic growth condition. Bioresour Technol.

[39] Haggerty JM, Dinsdale EA (2017). Distinct biogeographical patterns of marine bacterial taxonomy and functional genes. Glob Ecol Biogeogr.

[40] Handley KM, Lloyd JR (2013) Biogeochemical implications of the ubiquitous colonization of marine habitats and redox gradients by Marinobacter species. Front Microbiol 4: 1–10 10.3389/fmicb.2013.00136 PMC3660661 23734151

[41] Hazon N, Wells A, Pillans RD, Good JP, Gary Anderson W, Franklin CE (2003). Urea based osmoregulation and endocrine control in elasmobranch fish with special reference to euryhalinity. Comp Biochem Physiol B Biochem Mol Biol.

[42] Jeffree RA, Oberhansli F, Teyssie JL (2007). Accumulation and transport behaviour of 241americium, 60cobalt and 134cesium by eggs of the spotted dogfish Scyliorhinus canicula. Mar Pollut Bull.

[43] Jeffree RA, Oberhansli F, Teyssie JL (2008). The accumulation of lead and mercury from seawater and their depuration by eggs of the spotted dogfish Scyliorhinus canicula (Chondrichthys). Arch Environ Contam Toxicol.

[44] Jeffree RA, Oberhansli F, Teyssie JL (2010). Phylogenetic consistencies among chondrichthyan and teleost fishes in their bioaccumulation of multiple trace elements from seawater. Sci Total Environ.

[45] Jeffree RA, Warnau M, Oberhansli F, Teyssie JL (2006). Bioaccumulation of heavy metals and radionuclides from seawater by encased embryos of the spotted dogfish Scyliorhinus canicula. Mar Pollut Bull.

[46] Jeffree RA, Warnau M, Teyssie JL, Markich SJ (2006). Comparison of the bioaccumulation from seawater and depuration of heavy metals and radionuclides in the spotted dogfish Scyliorhinus canicula (Chondrichthys) and the turbot Psetta maxima (Actinopterygii: Teleostei). Sci Total Environ.

[47] Johri S, Doane MP, Allen L, Dinsdale EA (2019) Taking advantage of the genomics revolution for monitoring and conservation of chondrichthyan populations. Diversity 11

[48] Kanehisa M, Goto S, Sato Y, Furumichi M, Tanabe M (2012). KEGG for integration and interpretation of large-scale molecular data sets. Nucleic Acids Res.

[49] Kanehisa M, Sato Y, Kawashima M, Furumichi M, Tanabe M (2016). KEGG as a reference resource for gene and protein annotation. Nucleic Acids Res.

[50] Kim M, Lee S, Park HD, Choi SI, Hong S (2012). Biofouling control by quorum sensing inhibition and its dependence on membrane surface. Water Sci Technol.

[51] Kimes NE, Johnson WR, Torralba M, Nelson KE, Weil E, Morris PJ (2013). The Montastraea faveolata microbiome: ecological and temporal influences on a Caribbean reef-building coral in decline. Environ Microbiol.

[52] Koblizek M, Beja O, Bidigare RR, Christensen S, Benitez-Nelson B, Vetriani C, Kolber MK, Falkowski PG, Kolber ZS (2003). Isolation and characterization of Erythrobacter sp strains from the upper ocean. Arch Microbiol.

[53] Lang A (2020). The speedy secret of shark skin. Phys Today.

[54] Lidbury ID, Murrell JC, Chen Y (2015). Trimethylamine and trimethylamine N-oxide are supplementary energy sources for a marine heterotrophic bacterium: implications for marine carbon and nitrogen cycling. ISME J.

[55] Lima LFO, Weissman M, Reed M, Papudeshi B, Alker AT, Morris MM, Edwards RA, de Putron SJ, Vaidya NK, Dinsdale EA (2020) Modeling of the coral microbiome: the influence of temperature and microbial network. MBio 11:1–17 10.1128/mBio.02691-19 PMC7064765 32127450

[56] Lima N, Rogers T, Acevedo-Whitehouse K, Brown MV (2012). Temporal stability and species specificity in bacteria associated with the bottlenose dolphins respiratory system. Environ Microbiol Rep.

[57] Liu S, Liu Z (2020). Distinct capabilities of different Gammaproteobacterial strains on utilizing small peptides in seawater. Sci Rep.

[58] Llewellyn MS, Boutin S, Hoseinifar SH, Derome N (2014). Teleost microbiomes: the state of the art in their characterization, manipulation and importance in aquaculture and fisheries. Front Microbiol.

[59] Loomis KH, Wu SK, Ernlund A, Zudock K, Reno A, Blount K, Karig DK (2021). A mixed community of skin microbiome representatives influences cutaneous processes more than individual members. Microbiome.

[60] Lopez-Perez M, Gonzaga A, Martin-Cuadrado AB, Onyshchenko O, Ghavidel A, Ghai R, Rodriguez-Valera F (2012). Genomes of surface isolates of Alteromonas macleodii: the life of a widespread marine opportunistic copiotroph. Sci Rep.

[61] Lowery NV, Ursell T (2019). Structured environments fundamentally alter dynamics and stability of ecological communities. Proc Natl Acad Sci USA.

[62] Lynch JB, Hsiao EY (2019). Microbiomes as sources of emergent host phenotypes. Science.

[63] Lyons K, Lowe CG (2013) “Mechanisms of Maternal Transfer of Organochlorine Contaminants and Mercury in the Common Thresher Shark (Alopias Vulpinus).” Canad J Fish Aquat Sci 70(12):1667–72. 10.1139/cjfas-2013-022

[64] Magoc T, Salzberg SL (2011). FLASH: fast length adjustment of short reads to improve genome assemblies. Bioinformatics.

[65] Mason OU, Hazen TC, Borglin S, Chain PS, Dubinsky EA, Fortney JL, Han J, Holman HY, Hultman J, Lamendella R, Mackelprang R, Malfatti S, Tom LM, Tringe SG, Woyke T, Zhou J, Rubin EM, Jansson JK (2012). Metagenome, metatranscriptome and single-cell sequencing reveal microbial response to Deepwater Horizon oil spill. ISME J.

[66] Math RK, Jin HM, Kim JM, Hahn Y, Park W, Madsen EL, Jeon CO (2012). Comparative genomics reveals adaptation by Alteromonas sp. SN2 to marine tidal-flat conditions: cold tolerance and aromatic hydrocarbon metabolism. Plos One.

[67] Maz-Courrau A, Lopez-Vera C, Galvan-Magana F, Escobar-Sanchez O, Rosiles-Martinez R, Sanjuan-Munoz A (2012). 'Bioaccumulation and biomagnification of total mercury in four exploited shark species in the Baja California Peninsula, Mexico. Bull Environ Contam Toxicol.

[68] McMurdie PJ, Holmes S (2014). Waste not, want not: why rarefying microbiome data is inadmissible. Plos Comput Biol.

[69] Meyer F, Paarmann D, D'Souza M, Olson R, Glass EM, Kubal M, Paczian T, Rodriguez A, Stevens R, Wilke A, Wilkening J, Edwards RA (2008) The metagenomics RAST server - a public resource for the automatic phylogenetic and functional analysis of metagenomes. BMC Bioinformatics 9. 10.1186/1471-2105-9-386 PMC2563014 18803844

[70] Meyer W, Seegers U (2012). Basics of skin structure and function in elasmobranchs: a review. J Fish Biol.

[71] Meyer W, Seegers U, Stelzer R (2007). Sulphur, thiols, and disulphides in the fish epidermis, with remarks on keratinization. J Fish Biol.

[72] Mika K, Okamoto AS, Shubin NH, Welch DBM (2021) Bacterial community dynamics during embryonic development of the little skate (*Leucoraja erinacea*). Anim Microbiome 3 10.1186/s42523-021-00136-x PMC8513177 34645528

[73] Minich JJ, Morris MM, Brown M, Doane M, Edwards MS, Michael TP, Dinsdale EA (2018) Elevated temperature drives kelp microbiome dysbiosis, while elevated carbon dioxide induces water microbiome disruption. Plos One 13 10.1371/journal.pone.0192772 PMC5825054 29474389

[74] Moran MA, Belas R, Schell MA, Gonzalez JM, Sun F, Sun S, Binder BJ, Edmonds J, Ye W, Orcutt B, Howard EC, Meile C, Palefsky W, Goesmann A, Ren Q, Paulsen I, Ulrich LE, Thompson LS, Saunders E, Buchan A (2007). Ecological genomics of marine Roseobacters. Appl Environ Microbiol.

[75] Morris MM, Frixione NJ, Burkert AC, Dinsdale EA, Vannette RL (2020) Microbial abundance, composition, and function in nectar are shaped by flower visitor identity. FEMS Microbiol Ecol 96 10.1093/femsec/fiaa003 31922546

[76] Nosal AP, Caillat A, Kisfaludy EK, Royer MA, Wegner NC (2014). 'Aggregation behavior and seasonal philopatry in male and female leopard sharks Triakis semifasciata along the open coast of southern California, USA. Mar Ecol Prog Ser.

[77] Nosal AP, Cartamil DC, Long JW, Luhrmann M, Wegner NC, Graham JB (2013). 'Demography and movement patterns of leopard sharks (Triakis semifasciata) aggregating near the head of a submarine canyon along the open coast of southern California, USA. Environ Biol Fish.

[78] Ondov BD, Bergman NH, Phillippy AM (2011). Interactive metagenomic visualization in a Web browser. BMC Bioinforma.

[79] Overbeek R, Begley T, Butler RM, Choudhuri JV, Chuang HY, Cohoon M, de Crecy-Lagard V, Diaz N, Disz T, Edwards R, Fonstein M, Frank ED, Gerdes S, Glass EM, Goesmann A, Hanson A, Iwata-Reuyl D, Jensen R, Jamshidi N, Krause L, Kubal M, Larsen N, Linke B, McHardy AC, Meyer F, Neuweger H, Olsen G, Olson R, Osterman A, Portnoy V, Pusch GD, Rodionov DA, Ruckert C, Steiner J, Stevens R, Thiele I, Vassieva O, Ye Y, Zagnitko O, Vonstein V (2005). The subsystems approach to genome annotation and its use in the project to annotate 1000 genomes. Nucleic Acids Res.

[80] Peach MB (2005). New microvillous cells with possible sensory function on the skin of sharks. Mar Freshw Behav Physiol.

[81] Perry CT, Pratte ZA, Clavere-Graciette A, Ritchie KB, Hueter RE, Newton AL, Fischer GC, Dinsdale EA, Doane MP, Wilkinson KA, Bassos-Hull K, Lyons K, Dove ADM, Hoopes LA, Stewart FJ (2021). Elasmobranch microbiomes: emerging patterns and implications for host health and ecology. Anim Microbiome.

[82] Pogoreutz C, Gore MA, Perna G, Millar C, Nestler R, Ormond RF, Clarke CR, Voolstra CR (2019). Similar bacterial communities on healthy and injured skin of black tip reef sharks. Anim Microbiome.

[83] Quince C, Walker AW, Simpson JT, Loman NJ, Segata N (2017). Shotgun metagenomics, from sampling to analysis. Nat Biotechnol.

[84] Sabirova JS, Ferrer M, Regenhardt D, Timmis KN, Golyshin PN (2020). Proteomic insights into metabolic adaptations in Alcanivorax borkumensis induced by alkane utilization. J Bacteriol.

[85] Schlame M, Brody S, Hostetler KY (1993). Mitochondrial cardiolipin in diverse eukaryotes - comparison of biosynthetic reactions and molecular acyl species. Eur J Biochem.

[86] Schmieder R, Edwards R (2011) Fast identification and removal of sequence contamination from genomic and metagenomic datasets. Plos One 6 10.1371/journal.pone.0017288 PMC3052304 21408061

[87] Shadwick RE, Farrell AP, Brauner CJ, eds. (2015) Physiology of Elasmobranch Fishes: Internal Processes. Academic Press

[88] Speers-Roesch B, Treberg JR (2010). The unusual energy metabolism of elasmobranch fishes. Comp Biochem Physiol A: Mol Integr Physiol.

[89] Storo R, Easson C, Shivji M, Lopez JV (2021). Microbiome analyses demonstrate specific communities within five shark species. Front Microbiol.

[90] Sullivan T, Regan F (2011). The characterization, replication and testing of dermal denticles of Scyliorhinus canicula for physical mechanisms of biofouling prevention. Bioinspiration Biomimetics.

[91] Sullivan T, Regan F (2011b) The characterization, replication and testing of dermal denticles of *Scyliorhinus canicula* for physical mechanisms of biofouling prevention. Bioinspiration Biomimetics 6 10.1088/1748-3182/6/4/046001 21992932

[92] Sylvain FE, Holland A, Bouslama S, Audet-Gilbert E, Lavoie C, Val AL, Derome N (2020) Fish skin and gut microbiomes show contrasting signatures of host species and habitat. Appl Environ Microbiol 86: 1–15 10.1128/AEM.00789-20 PMC7414953 32503908

[93] Szczepaniak J, Press C, Kleanthous C (2020). The multifarious roles of Tol-Pal in Gram-negative bacteria. FEMS Microbiol Rev.

[94] Tahon G, Lebbe L, De Troch M, Sabbe K, Willems A (2020). Leeuwenhoekiella aestuarii sp. nov., isolated from salt-water sediment and first insights in the genomes of Leeuwenhoekiella species. Int J Syst Evol Microbiol.

[95] Tsutsui S, Dotsuta Y, Ono A, Suzuki M, Tateno H, Hirabayashi J, Nakamura O (2014). A C-type lectin isolated from the skin of Japanese bullhead shark (Heterodontus japonicus) binds a remarkably broad range of sugars and induces blood coagulation. J Biochem.

[96] Vendl C, Nelson T, Ferrari B, Thomas T, Rogers T (2021). Highly abundant core taxa in the blow within and across captive bottlenose dolphins provide evidence for a temporally stable airway microbiota. BMC Microbiol.

[97] Walsh K, Haggerty JM, Doane MP, Hansen JJ, Morris MM, Moreira APB, de Oliveira L, Leomil L, Garcia GD, Thompson F, Dinsdale EA (2017) Aura-biomes are present in the water layer above coral reef benthic macro-organisms. Peerj 5 10.7717/peerj.3666 PMC5562181 28828261

[98] Weitzman CL, Gibb K, Christian K (2018). Skin bacterial diversity is higher on lizards than sympatric frogs in tropical Australia. Peerj.

[99] Wen L, Thornycroft PJM, Weaver JC, Lauder GV (2015). Hydrodynamic function of biomimetic shark skin: effect of denticle pattern and spacing. Integr Comp Biol.

[100] Williams TJ, Ertan H, Ting L, Cavicchioli R (2009). Carbon and nitrogen substrate utilization in the marine bacterium Sphingopyxis alaskensis strain RB2256. ISME J.

[101] Wilson B, Danilowicz BS, Meijer WG (2008). The diversity of bacterial communities associated with Atlantic cod Gadus morhua. Microb Ecol.

[102] Yang L, Wang XH, Cui S, Ren YX, Yu J, Chen N, Xiao Q, Guo LK, Wang RH (2019). Simultaneous removal of nitrogen and phosphorous by heterotrophic nitrification-aerobic denitrification of a metal resistant bacterium Pseudomonas putida strain NP5. Bioresour Technol.

[103] Zhang DY, Li YY, Han X, Li XA, Chen HW (2011). High-precision bio-replication of synthetic drag reduction shark skin. Chin Sci Bull.

[104] Zheng Q, Lin WX, Liu YT, Chen C, Jiao NZ (2016) A comparison of 14 Erythrobacter genomes provides insights into the genomic divergence and scattered distribution of phototrophs. Front Microbiol 7 10.3389/fmicb.2016.00984 PMC4919336 27446024

