# Supplementary material for: The Epidermal Microbiome Within an Aggregation of Leopard Sharks (Triakis semifasciata) Has Taxonomic Flexibility with Gene Functional Stability Across Three Time-points
Source: Microb Ecol. 2022 Feb 7;85(2):747–64. doi: 10.1007/s00248-022-01969-y (PMC9957878; doi:10.1007/s00248-022-01969-y)
Supplement: Supplementary file 1 — Supplementary file1 (DOCX 504 KB) [file 248_2022_1969_MOESM1_ESM.docx]

**Supplementary material**


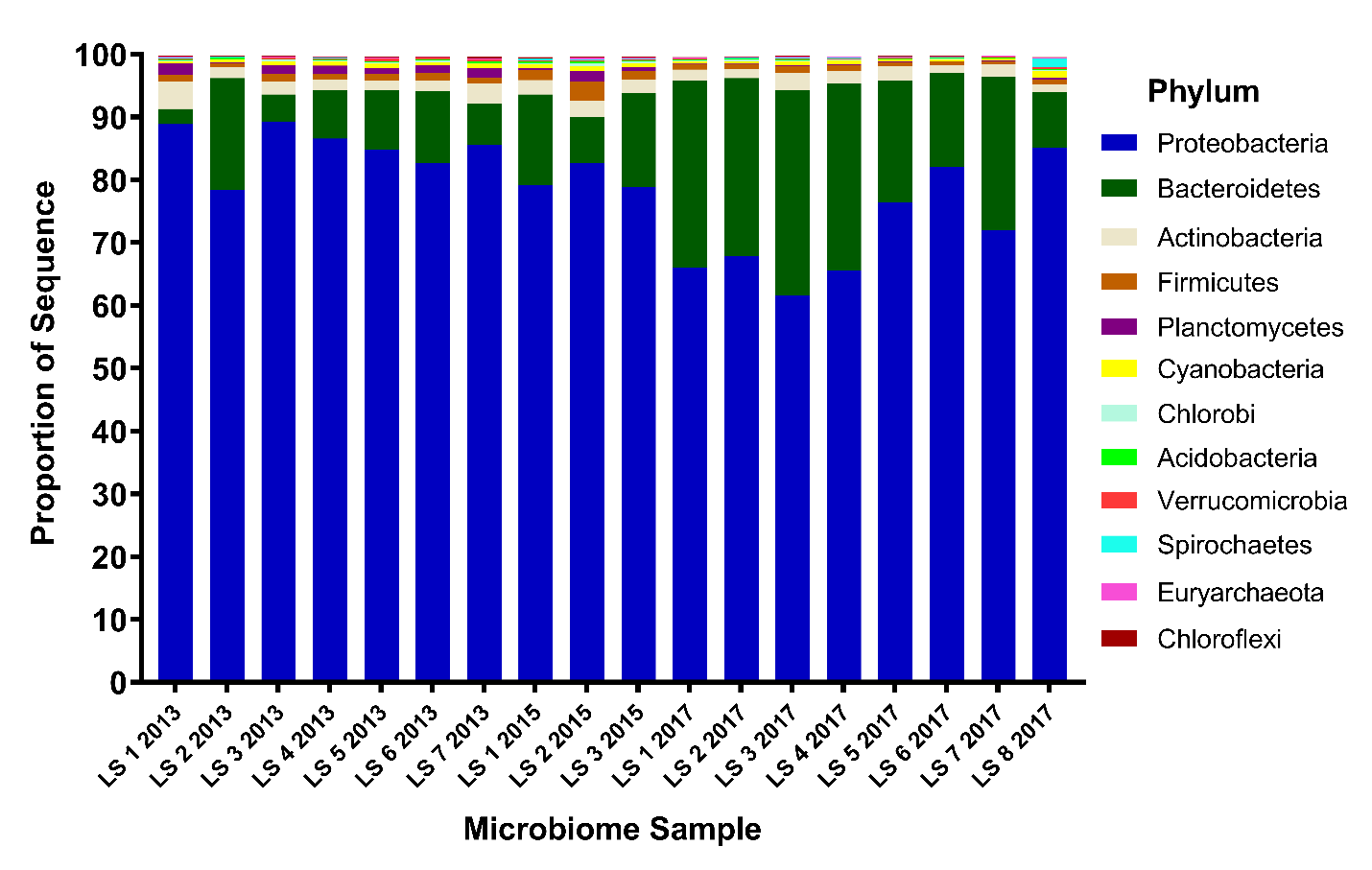


Supplementary Figure 1. The phylum profile of the *Triakis semifasciata* epidermal microbiome had stable across four years.


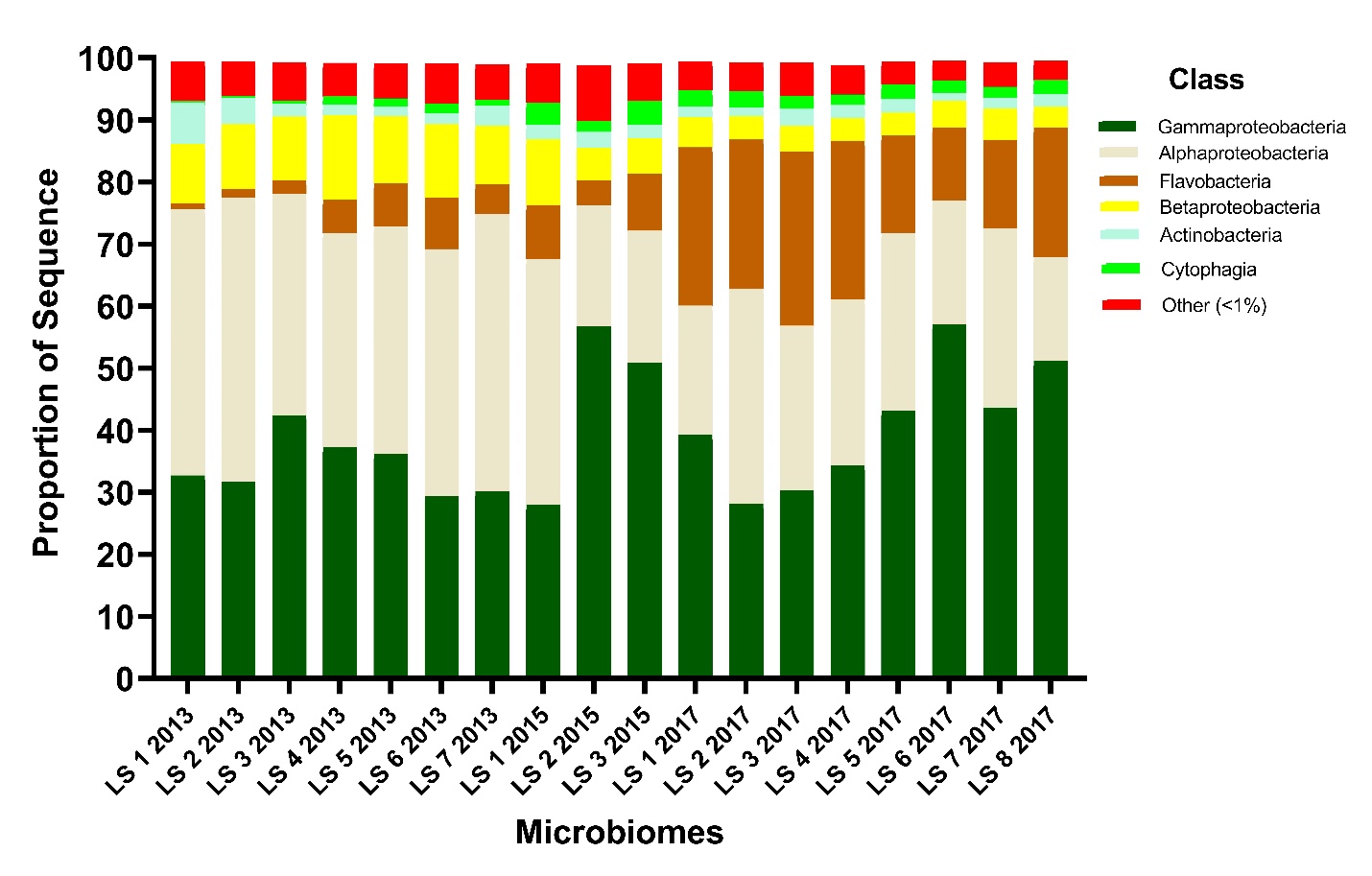


Supplementary Figure 2. The relative distribution of microbial classes in the *Triakis semifasciata* microbiome were similar across four years.


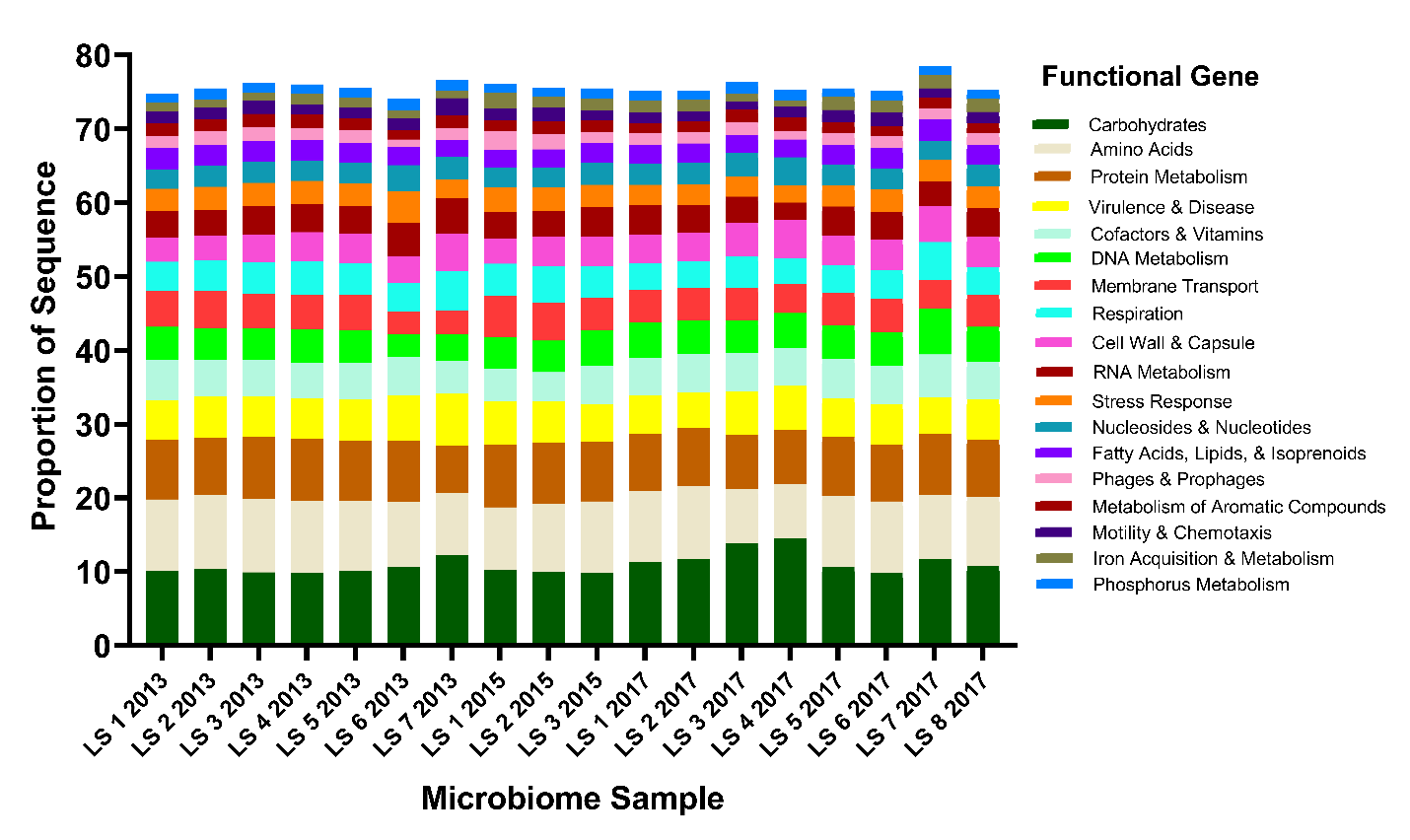


Supplementary Figure 3. The major metabolic processes identifying within the *Triakis semifasciata* microbiomes.
